# SGSS05-NS3, a covalent SETD8 inhibitor that activates p53 pathway in neuroblastoma

**DOI:** 10.1186/s13046-025-03565-7

**Published:** 2025-12-19

**Authors:** Zhihui Liu, Sukriti Bagchi, Chunhua Yan, Ying Hu, Gil Blum, Anqi Ma, Jian Jin, Minkui Luo, Sebastiano Di Bella, Francesco Verona, Ettore Appella, Giuseppe Giannini, Carol J. Thiele, Veronica Veschi

**Affiliations:** 1 Cell and Molecular Biology Section, Pediatric Oncology Branch, Center for Cancer Research, National Cancer Institute, CRC, 1-3940, 10 Center Drive MSC-1105, Bethesda, MD 20892 USA; 2 Center for Biomedical Informatics and Information Technology, Center for Cancer Research, National Cancer Institute, Rockville, MD USA; 3 Tri-Institutional PhD Program of Chemical Biology, Memorial Sloan Kettering Cancer Center, New York, NY 10065 USA; 4 Chemical Biology Program, Memorial Sloan Kettering Cancer Center, New York, NY 10065 USA; 5 Mount Sinai Center for Therapeutics Discovery, Departments of Pharmacological Sciences, Icahn School of Medicine at Mount Sinai, Oncological Sciences and Neuroscience, Tisch Cancer Institute, New York, NY 10029 USA; 6 Program of Pharmacology, Weill Cornell Medical College of Cornell University, New York, NY 10021 USA; 7 Department of Precision Medicine in Medical, Surgical and Critical Care, University of Palermo, Palermo, Italy; 8 Department of Health Promotion Sciences, Internal Medicine and Medical Specialties, University of Palermo, Palermo, 90127 Italy; 9 Chemical Immunology Section, Laboratory of Cell Biology, National Cancer Institute, Bethesda, MD USA; 10 Department of Molecular Medicine, University of Rome La Sapienza, Rome, 00161 Italy; 11 Istituto Pasteur-Fondazione Cenci Bolognetti, Rome, 00161 Italy

**Keywords:** Neuroblastoma, MYCN, P53 methylation, UNC0379, Topotecan, Bliss value, Synergy score

## Abstract

**Background:**

High-risk neuroblastoma (NB) is one of the most aggressive pediatric tumors accounting for 15% of all pediatric oncology deaths, and with less than 50% of patients experience long-term survival despite intense multimodal treatment. The tumor suppressor p53 is rarely (2%) mutated in NB but its functions are diminished in the majority of these tumors. Multiple mechanisms have been identified that attenuate the activity of p53 in MYCN-amplified (MYCN-amp) NB cells, but fewer mechanisms of p53 inactivation have been revealed in MYCN-WT NBs. Thus, a major challenge is to identify novel targeted therapies for high-risk NB (HR-NB) patients, specifically for the large fraction (70%) that present with MYCN-WT. Previously, we identified SETD8, the H4^K20me1^ methyltransferase, as a crucial epigenetic regulator of growth and differentiation in NB. In addition to targeting other non-histone proteins, SETD8 monomethylates p53 on lysine 382 (p53^K382me1^), attenuating its pro-apoptotic and growth arrest functions. Genetic and pharmacological (UNC0379) inhibition of SETD8 impairs NB growth *in vivo*.

**Methods:**

IC_50_ and IVTI (*in vitro* therapeutic index) of SGSS05-NS3, a SETD8 inhibitor, were measured in a broad collection of MYCN-WT and MYCN-amp NB cell lines. We took advantage of RNA-seq transcriptome analysis, *in vitro* functional assays and *in vivo* preclinical NB models.

**Results:**

To identify targeted therapies that are less toxic for HR-NB, we evaluated a more specific SETD8 inhibitor with enhanced activity and selectivity, SGSS05-NS3. Our results indicated that in NB cells *in vitro* treatment with SGSS05-NS3 rescues the canonical p53 functions leading to increases in p53 protein levels and of its target p21 by decreasing p53^K382me1^, impairing NB cell viability and inducing caspase-dependent cell death. Gene expression profile (RNA-seq analysis) confirmed that the most significantly upregulated genes upon SGSS05-NS3 treatment were among the p53 pathway targets. Pharmacological and genetic SETD8 inhibition restores p53-mediated DNA damage response. In pre-clinical xenograft NB models, pharmacological SETD8 inhibition by SGSS05-NS3 conferred a significant survival advantage in MYCN-WT NB.

**Conclusions:**

Our study provides further evidence for targeting SETD8 as a therapeutic strategy in NB, alone or in combination with Topotecan.

**Graphical Abstract:**

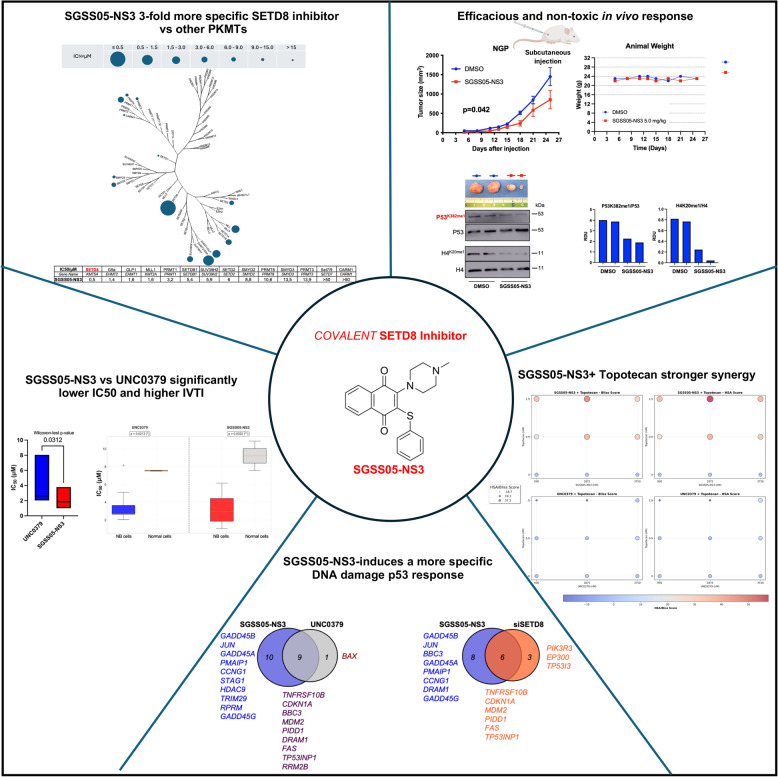

**Supplementary Information:**

The online version contains supplementary material available at 10.1186/s13046-025-03565-7.

## Background

The epigenetic machinery interprets cell intrinsic and extrinsic signals and in a dynamic, yet reversible process is recruited to specific genes to implement an ordered lineage-specifying program when a stem cell develops to a differentiated cell. Alterations in this epigenetic machinery (mutations or overexpression of many chromatin remodelers, DNA hypermethylation) lead to disruptions in normal development resulting in many pathologies, including cancer. Epigenetics and differentiation are intimately related in Neuroblastoma (NB), the most common extracranial solid tumor of childhood [1, 2]. Based on its histology, transcriptome analysis and its propensity to differentiate, NB has been considered a failure of the sympathoadrenal progenitors to differentiate [3]. High-risk NB (HR-NB) is one of the most aggressive pediatric tumors accounting for 15% of all pediatric oncology deaths, and with less than 50% of patients experience long-term survival, despite intense multimodal treatment [4].

In about 30% of HR-NBs, MYCN amplification represents the most powerful prognostic factor correlated with worse prognosis [5, 6]. As a transcription factor, MYCN has been historically considered “undruggable” despite several strategies aimed at targeting it are currently progressing [7]. *TP53* is rarely (< 2%) mutated in primary HR-NBs, but it is functionally impaired in both MYCN-amplified (MYCN-amp) and MYCN-WT NB tumors. While several mechanisms have been identified which inhibit the activity of p53 in MYCN-amp NB cells [8–10], fewer p53 inactivation mechanisms have been revealed in MYCN-WT NBs. Thus, a major challenge is to identify novel targeted therapies for the majority of HR-NB patients (70%) who present with MYCN-WT.

Given that few somatic mutations have been identified in NB patients (*ALK, PHOX2B*), the epigenome of NB tumors has recently been explored in order to identify critical enzymes as novel therapeutic targets for differentiation reprogramming. To uncover epigenetic machinery critical for maintenance of the undifferentiated and most aggressive NB phenotype, we identified histone methyltransferase SETD8 as an important and druggable NB dependency [11, 12].

SETD8 (*KMT5A*) specifically catalyzes mono-methylation of K20 on histone H4 (H4K20me1), implicated in mitotic progression, DNA replication, DNA damage response and chromosome condensation [13–18] In addition to targeting other non-histone proteins, SETD8 monomethylates p53 on lysine 382 (p53^K382me1^), attenuating its pro-apoptotic and growth arrest functions [19]. Moreover, H4K20me1 has been implicated in regulating the expression of p73, a member of the p53 family, and the DNA damage response via the regulation of 53BP1 [20–22].

Previously, we showed that SETD8 is overexpressed in NB cells compared to normal cells, and elevated SETD8 significantly correlates with poor prognosis in NB primary tumors, particularly the MYCN-WT subset [11]. Genetic and pharmacological (UNC0379) inhibition of SETD8 restores p53 canonical functions and impairs NB growth in *in vivo* preclinical models [11]. The chemical screen identified SETD8 inhibitor, UNC0379, as one of the most active and selective compounds in inhibiting NB cell growth [11]. Currently, there are few available substrate-competitive cell-permeable inhibitors targeting SETD8, among which is UNC0379 [23, 24]. To identify targeted therapy that is less toxic for HR-NB, we evaluated a more specific SETD8 inhibitor with enhanced activity and selectivity.

Here, we show that a more specific SETD8 inhibitor with enhanced activity and selectivity, SGSS05-NS3 rescues the canonical p53 functions, restores p53-mediated DNA damage response, and in pre-clinical xenograft NB models confers a significant survival advantage in MYCN-WT NB. This study provides further evidence for targeting SETD8 as a therapeutic strategy in NB, alone or in combination with Topotecan.

## Methods

### Cell lines and reagents

In this study, neuroblastoma (NB) cell lines were obtained from the cell line bank of the Pediatric Oncology Branch of the National Cancer Institute and have been genetically verified.

Five human MYCN-WT NB cell lines (SH-SY5Y (SY5Y), SK-N-AS (AS), NBEB, NBLS and SK-N-SH (SH)) and six human MYCN amplified NB cell lines (LAN1, SK-N-BE2C (BE2C), NGP, IMR32, SMS-KCNR (KCNR) and SMS-SAN (SAN)) were used in this study. NB cells were cultured in RPMI-1640 medium supplemented with 10% FBS, 2 mM L-glutamine, 100 μg/mL of penicillin/streptomycin at 37 °C in 5% CO2. ARPE-19 (human retinal pigment epithelial cell line), HEK293T (human embryonic kidney cell line) were obtained from the ATCC and cultured in DMEM/F12 with 10% fetal bovine serum. Cell cultures were tested and found to be mycoplasma-free.

Subconfluent cells were treated with SETD8 inhibitors, UNC0379 (kindly provided by Jian Jin), SGSS05-NS3 and SPECS21 (kindly provided by Minki Luo), at various concentrations for the indicated times.

For cell survival assays, NB cells were treated with different concentrations of SGSS05-NS3 (0.75, 1.5, 3 µM or 0.12, 0.37, 1.1, 3.3 and 10 µM). After 6, 12, 24 and 48 h of treatment, the number of viable cells and Trypan Blue-positive (non-viable) cells were counted.

Cell viability assay was conducted using the CellTiter 96® AQueous One Solution Cell Proliferation Assay (MTS, Promega) following the manufacturer's protocol. The results were analyzed with a GDV MPT reader (DV 990 BV6).

### Chemical screening in a 384-well format and epigenetic drugs

NB and control cell lines were plated in duplicate in 384-well plates at indicated densities (AS, 500 cells; NBEB and NBLS 2500 cells; NGP, 3500 cells; SAN, SY5Y, BE2C and KCNR 4000 cells; IMR32, 5000 cells; ARPE-19, 2000 cells; HEK293T, 3000 cells). The SETD8 inhibitors (UNC0379 and SGSS05-NS3) and Topotecan were dissolved in DMSO (or 95% ethanol) to a stock concentration of 10 mM and subsequently diluted to the indicated concentrations in cell culture media. After 24 h, cells were treated and cultured for the indicated time periods. Nine NB cell lines and two normal, immortal but non-transformed, cell lines were evaluated utilizing 12 doses for SETD8 inhibitors (0.1–30 μM) or 9 doses for Topotecan (0.1–30 nM) over the course of 7 days. The cell confluency was recorded every 4 h by IncuCyte Zoom System (Essen BioScience, Ann Arbor, MI, USA). The high-throughput chemical screening was performed three times. The average IC_50_ was calculated based on the cell confluency values of three biological replicates across nine NB cell lines after 96 h using the software GraphPad Prism 10. IC_50_ values are recorded as the natural logarithm of the half-maximal inhibitory µM concentrations. The *In Vitro* Therapeutic Index (IVTI) was calculated as the average IC_50_ of the control cell lines divided by the average IC_50_ of NB cell lines.

Average IC_50_ (µM) of the two epigenetic compounds SETD8 inhibitors, UNC0379 and SGSS05-NS3, in the indicated MYCN-WT (blue color) and MYCN-amplified (red color) NB cells compared with control cells at 72 h and 96 h along with the IVTI and p-values are shown in Table S1. Statistical significance for both IC_50_ values and IVTI was assessed using two-tailed Student’s t-tests and Wilcoxon test.

### Western blotting

Cells were washed 3 × in cold PBS, mechanically detached and the cell suspension divided in two with one harvested for total proteins and the other subjected to a histone protein extraction. Total protein extracts were obtained in RIPA buffer (50 mM Tris pH 8, 150 mM NaCl, 0.5% sodium deoxycholate, 0.1% SDS, 1% NP40, 1 mM EDTA and a mix of protease inhibitors). Total histone extracts were prepared using the EpiQuick Total Histone Extraction Kit according to the manufacturer’s protocol (Epigentek, Farmingdale, NY, USA).

Total protein extracts (30 μg/sample) and histone extracts (2 μg/sample) were separated by SDS-PAGE and blotted onto nitrocellulose membranes (PerkinElmer, Waltham, MA, USA). Membranes were blocked with 5% nonfat dry milk and incubated with primary antibodies (Abs) at the appropriate dilutions. The following Abs were used: mouse anti-p53 (DO-I), rabbit polyclonal anti-GAPDH and rabbit polyclonal anti-p21 (Santa Cruz Biotechnology, Santa Cruz, CA, USA); mouse anti-SETD8 (Abcam Inc, Cambridge, MA, USA); rabbit polyclonal anti-H4 and mouse anti-H4K20me1 (Active Motif, Carlsbad, CA, USA); rabbit polyclonal anti-PARP, rabbit monoclonal anti-53BP1 and rabbit monoclonal anti-ɣH2AX (Cell Signaling, Danvers, MA, USA); rabbit monoclonal anti-p53K382me1 (described previously [19]). Immunoreactive bands were visualized by enhanced chemiluminescence (Perkin Elmer). ECL signals were detected using film or Imager and were quantified using the Imager. Densitometry analysis was performed and measured as RDU (relative densitometric units) using Image J Software. All experiments were performed at least three times. The western blots shown are representative and associated densitometric analysis refer to the blots shown.

### Sample preparation for RNA-seq

RNA was isolated and subjected to RNA-seq analysis from SY5Y cells 12 h after treatment with DMSO or 1.5 µM (IC_50_) SGSS05-NS3, and from NGP cells 12 h after treatment with DMSO or 3 μM (IC_80_) SGSS05-NS3. Two independent biological replicates were performed. Total RNA extraction was carried out using a RNeasy Plus Kit (Qiagen Inc., Hilden, Germany) according to the manufacturer’s instructions. Transcriptional changes in SY5Y cells following 12 h of treatment with 1.5 µM (IC_50_) SGSS05-NS3 and in NGP cells following 12 h of treatment with 3 µM (IC_80_) SGSS05-NS3 were analyzed by RNA-seq (Illumina). This time was chosen as both histone and non-histone targets were inhibited and effects on cell viability were minimal.

#### Sequencing and alignment

Strand-specific whole transcriptome sequencing libraries were prepared using TruSeq® Stranded Total RNA LT Library Prep Kit (Illumina, San Diego, CA, USA) by following the manufacturer’s procedure. This protocol involved the removal of ribosomal RNA (rRNA) using biotinylated, target-specific oligos combined with Ribo-Zero rRNA removal beads. The RNA was fragmented into small pieces and the cleaved RNA fragments were reverse transcribed to generate first-strand cDNA using reverse transcriptase and random primers, followed by second-strand cDNA synthesis using DNA Polymerase I and RNase H. The resulting double-strand cDNA was used as the input to a standard Illumina library prep with end-repair, indexed adapter ligation and PCR amplification to generate sequencer-ready libraries. Eight indexed RNA-seq libraries were sequenced on a HiSeq2500 with Illumina TruSeq V4 chemistry (Illumina, San Diego, CA, USA). The Fastq files with 125 bp paired-end reads were processed using Trimmomatic (version 0.30) [25] to remove low quality bases. The trimmed fastq data were aligned to human genome hg19 with STAR (version 2.4.2a) [26] which used GENCODE gtf file version 19 (Ensembl 74). STAR software also generated the strand-specific gene read counts. About 77% of the 70 million reads per sample were mapped to the human genome uniquely for a total mapping rate of 90%.

#### Differential expression analysis

The gene read count data from STAR for SGSS05-NS3 samples were analyzed separately with R Package EdgeR (version 3.10.5) (Robinson et al., 2010). EdgeR performed the generalized linear model (GLM) likelihood ratio test to determine genes differentially expressed between any of the groups from each compound. EdgeR was also used to normalize the reads count data to generate z-scores for heatmap display.

#### Pathway analysis and heatmaps

Statistical results of differentially expressed genes from EdgeR were analyzed using QIAGEN’s Ingenuity® Pathway Analysis (IPA®, QIAGEN Redwood City, www.qiagen.com/ingenuity, IPA Fall Release, September 2015). Genes with p-values or false discovery rates (FDRs) less than the cutoff were used as input for IPA core analysis which calculated significant canonical pathways and generated pathway figures. Heatmaps for the top 50 up- or down-regulated genes for each compound were created in R using the heatmap.2 function in g plots (version 2.17.0).

### Real-Time PCR analysis

Total RNA extraction was carried out using an RNeasy Plus Kit (Qiagen Inc., Hilden, Germany) and quantitative reverse transcription-PCR (qRT-PCR) was performed as described [27]. The following primer sequences were used:

- CDKN1A_For AGGTGGACCTGGAGACTCTCAG,
- CDKN1A_Rev TCCTCTTGGAGAAGATCAGCCG,
- FAS_For TGAAGGACATGGCTTAGAAGTG,
- FAS_Rev CGTGCAAGGGTCACAGTGTT,
- GADD45A_For CTGGAGGAAGTGCTCAGCAAAG,
- GADD45A_Rev AGAGCCACATCTCTGTCGTCGT,
- GADD45B_For GCCAGGATCGCCTCACAGTGG,
- GADD45B_Rev GGATTTGCAGGGCGATGTCATC.

### Animal experiments

All xenograft studies were approved by the Animal Care and Use Committee of the National Cancer Institute, and all mouse treatments, including their housing, were in accordance with the institutional guidelines (PB-023).

For subcutaneous injection, SY5Y cells were washed with Hanks balanced salt solution (HBSS) (Invitrogen), and re-suspended in HBSS and Matrigel (Trevigen, Gaitherburg, MD, USA). Cell suspension (100 μl) containing 2 × 10^6^ cells was inoculated into the subcutaneous tissue of the left flank of 5- to 6-week-old female athymic nude mice (Taconic, Germantown, NY) using a 28-gauge needle (Becton Dickinson, Franklin Lakes, NJ). For the ex-vivo pharmacological inhibition of SETD8, SY5Y cells were treated *ex-vivo* with 1.5 µM of SGSS05-NS3 or DMSO for 24 h and then injected into nude mice (10 mice per group). For the continuous *in vivo* pharmacological inhibition of SETD8, NGP cells were injected into nude mice. Mice were treated with 5 mg/kg of SGSS05-NS3 compound i.p. three times a week for three weeks (6 mice per group). To avoid any gender-related bias the NGP cells were injected into nude male mice.

The dimensions, length (L) and width (W), of the resulting tumors were determined three times per week using a digital caliper, and the tumor volume (mm3) was calculated as (L × W^2^)/4. Twenty-five days after the injection 5 tumors from each group were excised and tissue samples from these were used to assess SETD8 expression and its targets.

### Immunohistochemistry and Immunofluorescence

Immunohistochemical analysis was performed on 4 µm-thick paraffin-embedded sections derived from xenograft tumor samples generated by the subcutaneous injection of SY5Y cells untreated or treated *ex-vivo* with 1.5 µM of SGSS05-NS3 as previously reported and subsequently exposed to specific antibodies against SETD8 (Abcam Inc, Cambridge, MA, USA) and p53K382me1 (described previously [19]. Then, antigens were revealed using polymer horseradish peroxidase (HRP) conjugated antibodies (Biocare Medical) and were detected by DAB and Vulcan Fast Red chromogen. The counterstaining of nuclei was performed with aqueous hematoxylin (Sigma-Aldrich) as described in the standard protocol. H&E stainings were performed using standard protocols.

Subconfluent cells were plated on 8 well chamber slide and treated with UNC0379 (1,2,4,8 µM) for 24 h or transfected with siSETD8 for 72 h. Indirect immunofluorescence was used to study the induction of ɣH2AX foci in SY5Y cells upon SGSS05-NS3 treatment. Cells were fixed in 4% formaldehyde/PBS for 15 min, permeabilized in 0.25% Triton/PBS for 10 min, blocked by incubation with 5% goat serum in PBS containing 0.1% Triton X-100 for 1 h RT, and incubated overnight with primary Abs, rabbit monoclonal anti-ɣH2AX and rabbit monoclonal anti-53BP1 (Cell Signaling, Danvers, MA, USA), dilution 1:100, followed by secondary FITC-conjugated species-specific antisera dilution 1:250 (Invitrogen). DAPI was added at a dilution of 1:10,000 (Invitrogen). Increased expression of two DNA damage response markers, ɣH2AX and 53BP1 was detected upon genetic and pharmacologic SETD8 inhibition with UNC0379 and SGSS05-NS3 treatment. Representative images of ɣH2AX expression are shown.

### Transfection, transduction and constructs

#### siRNA-mediated knockdown of SETD8

SETD8 knockdown was obtained by transfection of SY5Y cells for 72 h with two Dharmacon on-target plus siRNAs targeting human SETD8 (5'-GCAACUAGAGAGACAAAUC-3'/5'-GAUUGAAAGUGGGAAGGAA-3') using Lipofectamine 2000 reagent (Invitrogen) according to the manufacturer’s instructions. On-target plus non-targeting siRNA (5'-UGGUUUACAUGUCGACUAA-3', Dharmacon) was used as a control.

#### Short hairpin RNA (shRNA)-mediated knockdown of SETD8

HEK-293 T cells were transfected in OPTIMEM (Gibco) supplemented by XtremeGENE HP DNA transfection reagent (Roche), with TRIPZ inducible lentiviral nonsilencing shRNA control (ns shRNA, Dharmacon) or human SETD8 shRNA (shSETD8, Dharmacon), plasmids in association with psPAX2 (Addgene, 12,260) and pMD2.G (Addgene, 12,259) to produce lentiviral particles. SY5Y were transduced with LENTIX-Concentrator (cat#631,232) concentrated virus in presence of 8 μg/mL of polybrene (Sigma-Aldrich). Transduced cells were treated with doxycycline (1 μg/ml, Sigma-Aldrich) for 5–10 days.

LAN1 cells were transfected with p53 WT and p53K382R mutant TRIPZ inducible lentiviral plasmids (provided by GenScript), and SK-N-SH cells were transfected with empty (PLOC) or PLOC SETD8 (Dharmacon) inducible lentiviral plasmids, using Lipofectamine 2000 reagent (Invitrogen) according to the manufacturer’s instructions. Transfected cells were treated with doxycycline (1 μg/ml, Sigma-Aldrich) for 48 h. After 48 h cells were treated with or without 2 µM SGSS05-NS3 for 24 h. The empty pTRIPZ or PLOC vector was used as mock control.

### Bliss and HSA synergy score

To evaluate the synergistic effects of the combinatorial treatments in NB cell lines, synergy scores including Bliss and HSA, were calculated by using the SynergyFinder online tool [28]. Specifically, twelve doses of the SETD8 inhibitors UNC0379 and SGSS05-NS3 (0.1–30 μM) were combined with nine doses of Topotecan (0.1–30 nM) and analyzed over the course of 7 days. The cell confluency values of three biological replicates across five NB cell lines (NBLS, SY5Y, SAN, KCNR, and IMR32) were recorded every 4 h up to 96 h by IncuCyte Zoom System (Essen BioScience, Ann Arbor, MI, USA). Scores based on cell confluence data of three independent experiments, along with Bliss values obtained from combinations of Topotecan (nM) and SETD8 inhibitors SGSS05-NS3 or UNC0379 (µM) in five NB cell lines (NBLS, SY5Y, SAN, KCNR, and IMR32) at 48 h, 72 h, and 96 h are summarized in Table S3. P-values for Synergy scores (Bliss and HSA) were calculated by using t-tests, following SynergyFinder guidelines.

The degree of synergy or antagonism was quantified by comparing the observed drug combination responses against expected responses calculated using reference models that assume no interaction between drugs. The Bliss model assesses the multiplicative effect of single drugs acting independently, while the HSA model quantifies synergy as the excess over the maximum single-drug response. In particular, HSA model is one of the simplest reference models, which states that the expected combination effect is the maximum of the single drug responses at corresponding concentrations. Thus, HSA synergy score, S_HSA_, is defined as:

$$S_{HSA}=E_{A,B,...,N}-max\left(E_A,\;E_B,...,E_N\right),$$

where *E*_*A,B,…,N*_ is the combination effect between N drugs and *E*_*A*_*, E*_*B*_*,…, E*_*N*_ are the measured responses of the single drugs. While the Bliss independence model assumes a stochastic process in which two drugs elicit their effects independently, and the expected combination effect can be calculated based on the probability of independent events. Thus, Bliss synergy score, S_Bliss_, is defined as:

$$S_{Bliss}=E_{A,B,...N}-100\left(1-\left(1-E_A100\right)\left(1-E_B100\right)...\left(1-E_N100\right)\right),$$

Here, terms *(1—E*_*A,B,…,N*_*100)* represent probabilities that drugs *A,B,…,N* do not inhibit the target. Thus, multiplication of these terms results in the probability that none of the drugs inhibit the target. Subtracting this probability from 1, *1—(1—E*_*A*_*100)(1—E*_*B*_*100)…(1—E*_*N*_*100))*, gives the probability that at least one of the drugs inhibits the target, which is equivalent to the Bliss effect. A synergy score, Bliss or HSA, between 10 and −10 indicates that the interaction between two drugs is additive. A synergy score, Bliss or HSA, greater than 10 represent synergy. Conversely, scores below − 10 signify antagonism. Scores of 0 indicate no interaction.

### Statistical analysis

Statistical analyses were performed using Microsoft Excel, standard two-tailed Student’s *t* test and the software GraphPad Prism 10. Image J software was used for quantification of selected immunoblots.

For all cell survival and confluence assays, each experiment was performed at least three times. Statistical analysis was performed by a standard two-tailed Student’s *t* test. Statistical significance for both IC_50_ values and IVTI was assessed using two-tailed Student’s t-tests and Wilcoxon test. P-values for Synergy scores (Bliss and HSA) were calculated by using t-tests, following SynergyFinder guidelines.

Student’s t test was also used to compare the tumor volume between the SGSS05-NS3 and non-treatment groups, with *p*-values of < 0.05 being considered statistically significant. A paired t-test was used to compare the slopes of the growth rates. The analyses were carried out using the software GraphPad Prism 10.

The Kaplan–Meier method was used to determine the probability of mice survival as a function of time. The statistical significance between two treatment groups was evaluated using a log-rank test (Mantel- Cox). All *p*-values of < 0.05 were considered statistically significant. The analyses were carried out using the software GraphPad Prism 10.

## Results

### SGSS05-NS3, a covalent SETD8 inhibitor impairs NB cell growth regardless MYCN status

**Fig. 1:**
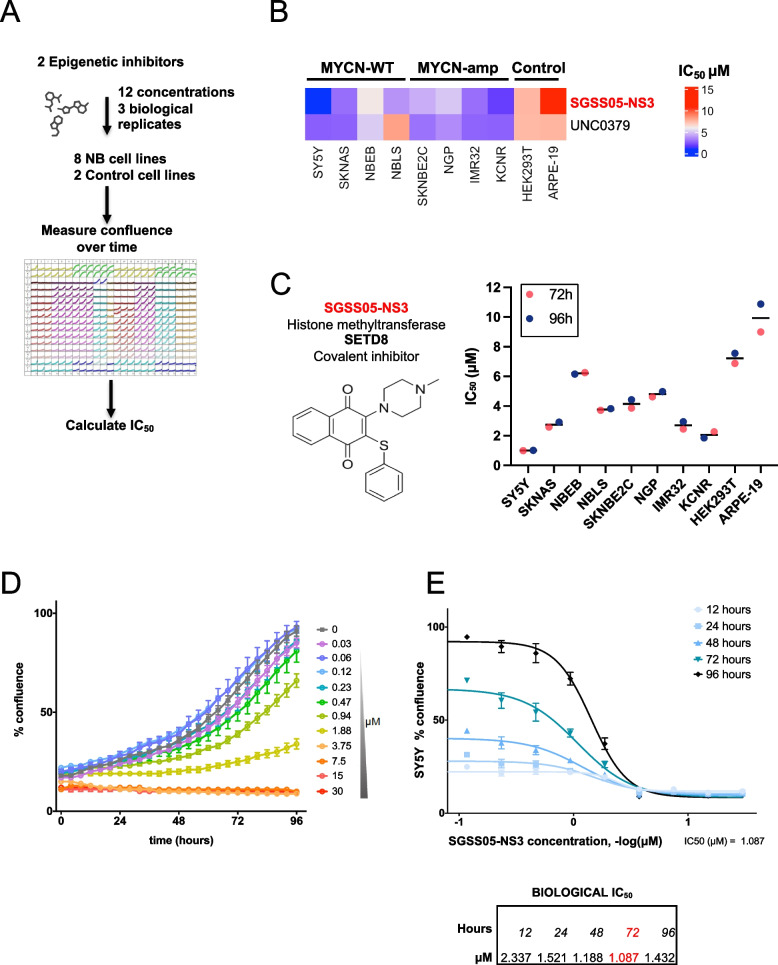
SGSS05-NS3, a covalent SETD8 inhibitor, impairs NB cell growth regardless MYCN status. **A** Flow chart showing the chemical screen methodology: 2 epigenetic compounds, SETD8 inhibitors UNC0379 and SGSS05-NS3, were tested in 8 NB cell lines (SY5Y, SKNAS, NBEB, NBLS, SKNBE2C, NGP, IMR32, KCNR) and 2 control cell lines (HEK293T and ARPE) to evaluate IC_50_ values. Cell confluence over time was recorded by an IncuCyte Zoom System. **B** Heatmap showing the average IC_50_ of the two SETD8 inhibitors, UNC0379 and SGSS05-NS3 at 96 h in 4 MYCN-WT (SY5Y, SKNAS, NBEB, NBLS) and 4 MYCN-amp (SKNBE2C, NGP, IMR32, KCNR), NB cell lines compared to 2 control cell lines (HEK293T and ARPE). The color key represents the average IC_50_ (µM) values: blue color represents low IC_50_ whereas red color indicates high IC_50_. The average IC_50_ was measured based on the cell confluence values of 3 biological replicates across 8 NB cell lines at 96 h using GraphPad Prism. For *in vitro* therapeutic index (IVTI) and p-values, see Table S1. **C** Average IC_50_ values of SGSS05-NS3 compound calculated as described in (B) in the indicated NB cell lines and control cells, at the indicated time points (average of 3 biological replicates). **D**, **E** An illustrative experiment showing SGSS05-NS3 treatment *in vitro* effects on cell viability at indicated time points (**D**) and concentrations (**E**) in SY5Y NB cells. Bars show the average of 3 replicates ± SD

Previously, by integrating a high-throughput siRNA screen and a chemical screen, we identified the H4^K20me1^ methyltransferase SETD8 as a crucial regulator of cell proliferation and differentiation in NB cells [11]. Moreover, we observed that genetic and pharmacological inhibition of SETD8, by using a substrate-competitive inhibitor, UNC0379 [23], impaired NB growth in *in vitro* assays and in preclinical *in vivo* models of NB. In order to identify less toxic targeted therapies for high-risk NB (HR-NB), we evaluated a covalent SETD8 inhibitor with enhanced activity, SGSS05-NS3 patented in 2015 (M. Luo, G. I. Sanchez, G. J. Blum, L. Yang, Sloan-Kettering Institute for Cancer Research, USA, Int. PCT Pub. No. WO2015172076 A1, 2015) in 8 NB cell lines (4 MYCN-WT and 4 MYCN-amp) and 2 control cell lines (HEK293T and ARPE-19) using 12 concentrations (0.03–30 µM). Cell viability was assessed over time up to 7 days and average IC_50_ was measured up to 96 h (Fig. 1A-C). SGSS05-NS3 at 3.2 µM exhibited average IC_50_ at 96 h and a highly significant p-value for the *In Vitro* Therapeutic Index (IVTI) across the 8 NB cell lines compared with control cell lines (Figure S1A-S1C and Table S1). A representative experiment illustrating SGSS05-NS3 effects on NB cell growth is shown (Fig. 1D and E). SGSS05-NS3 treatment was evaluated along with other two SETD8 inhibitors such as UNC0379 and SPECS21, showing lower IC_50_ across 5 NB cell lines (Figures S1D-S1E).

These findings demonstrate that SGSS05-NS3, a covalent SETD8 inhibitor impairs cell growth in both MYCN-WT and MYCN-amp NB cells, regardless MYCN status and without toxic effects in normal healthy cells.

### SGSS05-NS3 compound restores p53 functions by decreasing p53^K382me1^ and induces p53-dependent cell death in NB cells

Given that SETD8 monomethylates p53 on lysine 382 (p53^K382me1^), attenuating its pro-apoptotic and growth arrest functions [19], we evaluated p53 expression levels and its canonical activity in SY5Y cells upon SGSS05-NS3 treatment.

**Fig. 2:**
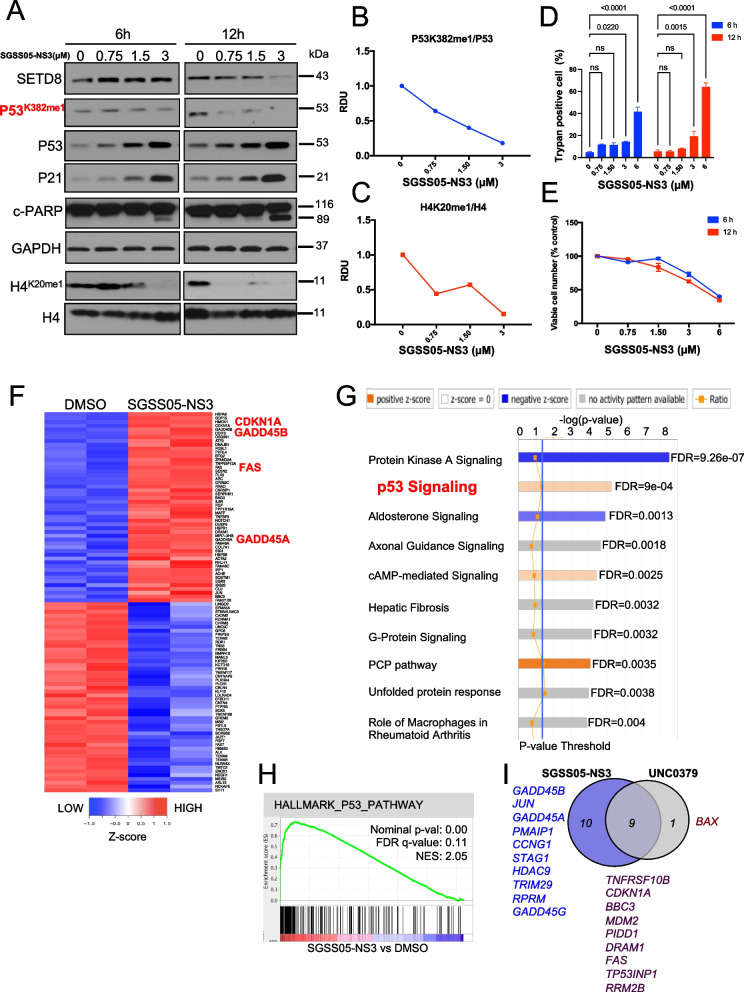
SGSS05-NS3 treatment reduces cell viability and induces p53 proapoptotic and growth arrest functions in NB cells. **A** Immunoblot analysis of the indicated total and histone proteins in SY5Y NB cells treated with SGSS05-NS3 at the indicated time points and concentrations. **B**, **C** Densitometry analysis of p53^K382me1^ protein levels normalized to p53 protein levels (**B**) and of H4^K20me1^ levels normalized to H4 protein levels (**C**) after treatment with the SETD8 inhibitor, SGSS05-NS3, for 12 h calculated as relative density units (RDU) using ImageJ software. **D**, **E** Trypan positive cell percentage (**D**) and viable cell number (**E**) in SY5Y NB cells treated with SGSS05-NS3 at the indicated time points and concentrations. Data are expressed as mean ± SD of three independent experiments. Statistical significance was calculated using the t test. ns, not significant. **D** Data are presented as % over control ± SD of three independent experiments (**E**). **F** Heatmap of the top 50 up- and down-regulated genes in SY5Y NB cells ranked by statistical significance following 12 h of treatment with 3 μM (IC_80_) SGSS05-NS3. Data are presented as normalized expression values of two biological replicates based on edgeR software analysis and FDR < 0.001. The color key represents the normalized expression values: blue (low) to red (high). **G** The top ten differentially expressed canonical pathways after SETD8 pharmacological inhibition by SGSS05-NS3 treatment in SY5Y cells treated as in (**F**), defined by Ingenuity Pathway Analysis (IPA) based on edgeR software analysis and FDR < 0.001. Pathways related to p53 signaling are among the top differentially expressed pathways. **H** Gene set enrichment analysis (GSEA) of the p53 downstream pathway (nominal *p* = 0.00, FDR = 0.11, normalized enrichment score [NES] = 2.05) upon SGSS05-NS3 treatment. See Table S2 for P53 downstream pathway gene list enriched. **I** Venn diagram of common (*n* = 9) and exclusive upregulated p53 target genes upon 3 μM SGSS05-NS3 and 4 μM UNC0379 treatment, respectively, for 12 h in SY5Y MYCN-WT NB cells

A dose-dependent decrease in the expression levels of H4^K20me1^ and p53^K382me1^, the histone and non-histone target of SETD8, respectively, was accompanied by enhanced p21 and p53 total protein levels (Fig. 2A-C and Figures S2A-S2C). Moreover, SGSS05-NS3 treatment led to a dose-dependent increased cell death (Trypan blue positive cells), reduced cell viability (Fig. 2D and E and Figure S2D), and increased levels of cleaved PARP (Fig. 2A and Figure S2A).

From RNA-seq data analysis emerged that p53-related targets were among the most significantly upregulated genes after treatment with SGSS05-NS3 compound. Specifically, *CDKN1A* (p21), *GADD45B*, *GADD45A* and *FAS* resulted among the top differentially upregulated genes, ranked by statistical significance (FDR < 0.001) (Fig. 2F and Figures S2E-S2G, and Table S2). Ingenuity Pathway Analysis (IPA) analysis of the differentially expressed genes identified the p53 signaling pathway among the most significantly up-regulated (Fig. 2G), and Gene Set Enrichment Analysis (GSEA) indicated enrichment in p53 downstream pathway (Fig. 2H). Interestingly, about 50% (9/20) of the differentially expressed p53 target genes upon SGSS05-NS3 treatment, overlapped with those induced by UNC0379 or by SETD8 silencing (6/17), including genes involved in the induction of cell death or cell apoptosis particularly in response to DNA damage, such as *PIDD1, TNFRSF10B, TP53INP1* and *FAS,* and genes related to growth arrest signals such as *CDKN1A* (p21) (Fig. 2I and Figure S2H). Of note, SGSS05-NS3 treatment compared with UNC0379 or with the SETD8 genetic inhibition, specifically activates *GADD45A*, *GADD45B* and *GADD45G*, p53 target genes involved in growth arrest, apoptosis and DNA-damage response (Fig. 2I and Figure S2H), confirming an enhanced activity of SGSS05-NS3 at increasing p53 total protein levels. The upregulation of the expression of selected genes upon SGSS05-NS3 treatment was confirmed (Figures S3A-S3D).

**Fig. 3:**
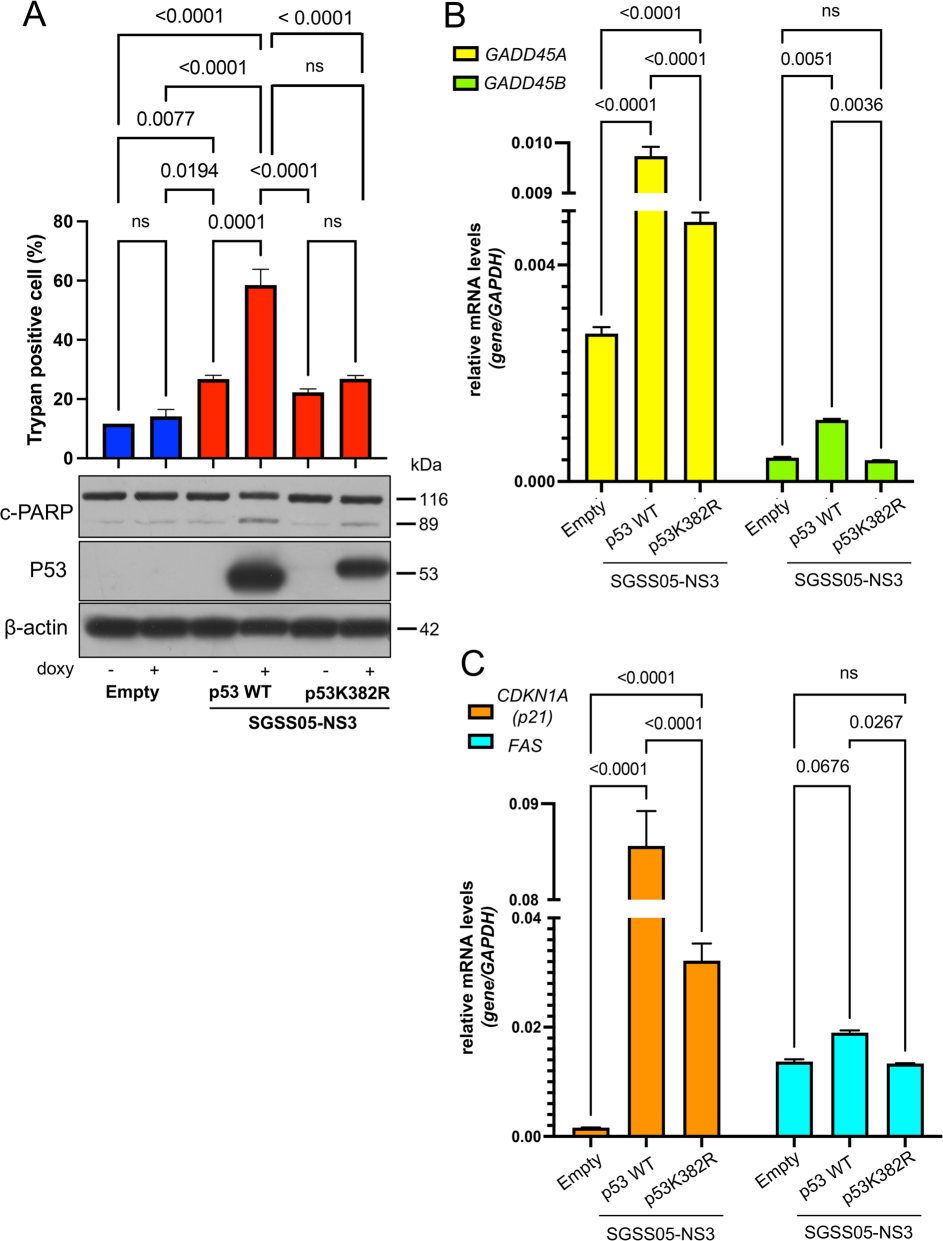
SETD8 inhibition by SGSS05-NS3 compound induces p53-dependent cell death in NB cells. **A** Trypan positive cell (%) and immunoblot analysis of the indicated proteins in LAN1, p53-null NB cells, upon transfection with p53 WT or p53K382R, treated with SGSS05-NS3 2 μM for 24 h. Bars show the mean ± SD of three replicates. ns, not significant. **B**, **C** Relative mRNA expression levels of *GADD45A, GADD45B, CDKN1A* (p21) and *FAS* in LAN1 upon transfection with p53 WT or p53K382R, treated with SGSS05-NS3 2 μM for 24 h. Bars show the mean ± SD of three replicates. ns, not significant

To investigate the causal role of p53 activation in response to SGSS05-NS3, p53-null NB cells LAN1 were stably transfected with doxycycline (doxy) inducible plasmids encoding for either WT or mutant p53, p53K382R (that cannot be methylated or acetylated), and subsequently treated with SGSS05-NS3 compound. Genetic rescue experiments showed that WT p53, but not mutant p53K382R, mediates an increased SGSS05-NS3-induced cell death accompanied by enhanced mRNA levels of p53 pro-growth arrest and pro-apoptotic target genes such as *GADD45A, GADD45B, CDKN1A* (p21) and *FAS* (Fig. 3A-C). These findings indicate that SETD8 inhibition via SGSS05-NS3 treatment induces a p53-dependent cell death.

All together, these data demonstrate that SETD8 inhibition via SGSS05-NS3 treatment impairs NB cell viability, induces a p53-dependent and caspase-mediated cell death, restoring p53 proapoptotic and growth arrest functions.

### SGSS05-NS3activates the p53 pathway via SETD8 inhibition in NB cells

**Fig. 4:**
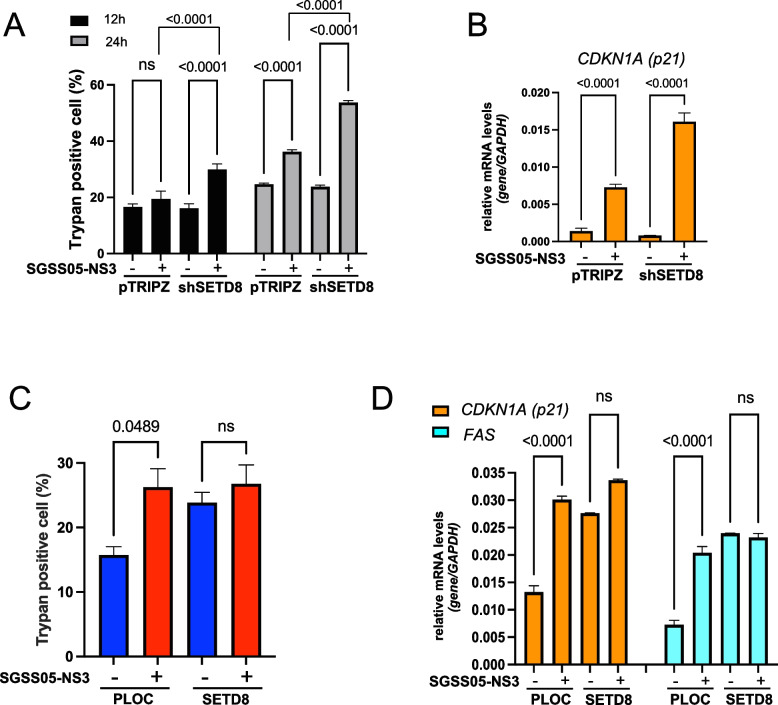
SGSS05-NS3 activates the p53 pathway via SETD8 inhibition in NB cells.** A** Trypan positive cell (%) in SY5Y NB cells upon transfection with pTRIPZ (empty vector) or shSETD8, treated with SGSS05-NS3 2 μM for 12 h and 24 h. Bars show the mean ± SD of three replicates. ns, not significant. **B** Relative mRNA expression levels of *CDKN1A* (p21) in SY5Y NB cells upon transfection with pTRIPZ (empty vector) or shSETD8, treated with SGSS05-NS3 2 μM for 24 h. Bars show the mean ± SD of three replicates. **C** Trypan positive cell (%) in SK-N-SH NB cells upon transfection with PLOC (empty vector) or PLOC SETD8 (SETD8), treated with SGSS05-NS3 2 μM for 24 h. Bars show the mean ± SD of three replicates. ns, not significant. **D** Relative mRNA expression levels of *CDKN1A* (p21) and *FAS* in SK-N-SH NB cells upon transfection with PLOC (empty vector) or PLOC SETD8, treated with SGSS05-NS3 2 μM for 24 h. Bars show the mean ± SD of three replicates. ns, not significant

Next, we conducted a detailed mechanistic investigation into the contribution of SETD8 in regulating the p53 pathway upon SGSS05-NS3 treatment in NB cells. We used a short hairpin lentiviral doxycycline-inducible vector to specifically target SETD8 expression (shSETD8) obtaining an 80% downregulation of SETD8 expression, and a PLOC lentiviral doxycycline-inducible vector overexpressing SETD8 levels up to 60% increased levels (Figures S4A and S4B). Treatment with SGSS05-NS3 significantly increased cell-death and the expression of p53 target genes upon SETD8 knockdown, suggesting the crucial role of SETD8 inhibition mediated by SGSS05-NS3 in reactivating p53 pro-apoptotic and growth arrest functions (Fig. 4A and B, and S4C).

SETD8 knockdown potentiates the effects of SGSS05-NS3 treatment in inducing cell death, whereas SETD8 overexpression counteracts these effects by impeding an increased expression of p53 target genes as *CDKN1A (p21)* and *FAS* upon SGSS05-NS3 treatment (Fig. 4C and D).

These findings support and validate our conclusions that SGSS05-NS3 activates the p53 pathway via SETD8 inhibition.

### SETD8 pharmacological inhibition upon SGSS05-NS3 treatment impairs tumor growth and prolongs murine survival in preclinical *in vivo *models of NB

**Fig. 5:**
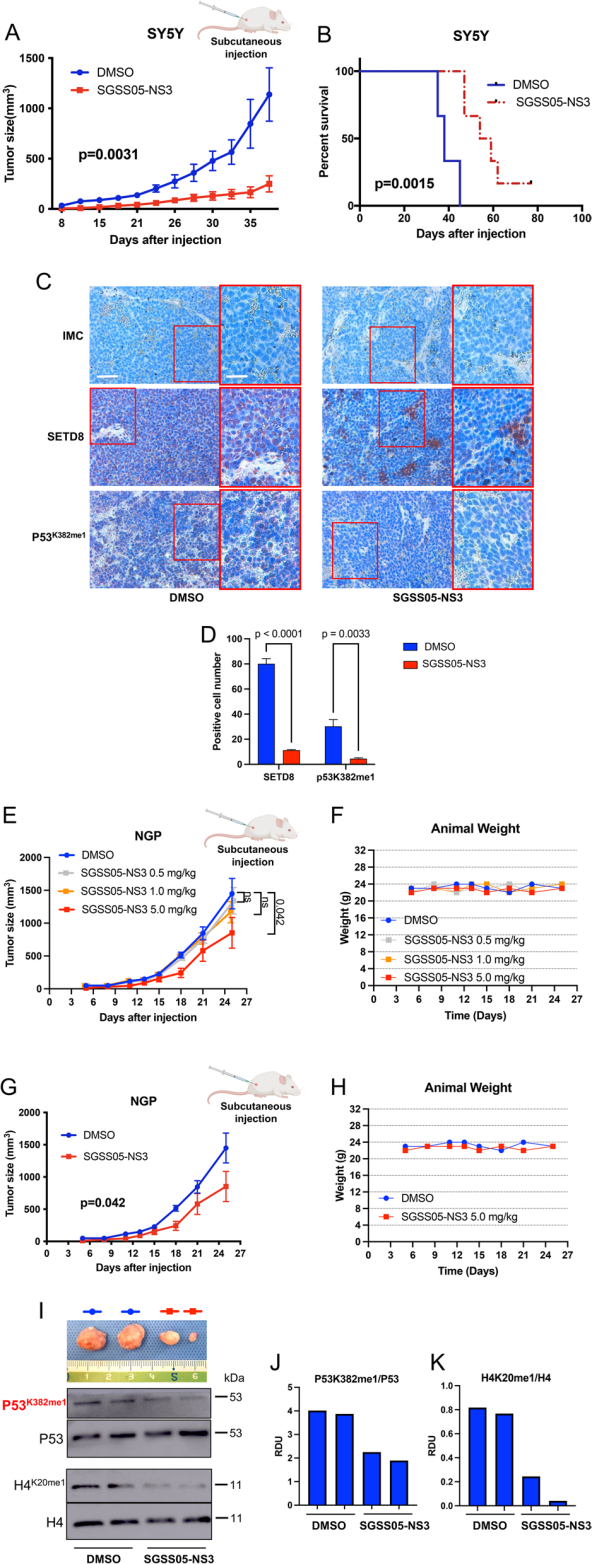
SETD8 pharmacological inhibition upon SGSS05-NS3 treatment impairs tumor growth and prolongs murine survival in preclinical *in vivo* models of NB. **A** SY5Y cells were treated *ex-vivo* with 1.5 µM SGSS05-NS3 for 24 h and then injected into nude mice. Day 0 indicates the day of cell injection. Bars show the tumor size average of 15 mice/group ± SEM. Slopes of the growth rate were compared by t test. **B** Kaplan-Meyer graph showing the murine survival after ex-vivo treatment of SY5Y with SGSS05-NS3 compound. The statistical significance between two treatment groups was evaluated using a log-rank test. **C** Immunohistochemical analysis of SETD8 and p53^K382me1^ on two tumors randomly chosen from each group (untreated and *ex-vivo* SGSS05-NS3 treated) collected 20 days after the injection. IMC = isotype matched control. Scale bars, 40 µm. **D** Positive cell number expressing SETD8 or p53^K382me1^ in two tumors randomly chosen from each xenograft group, generated by the injection of SY5Y NB cells, untreated (DMSO) or ex-vivo SGSS05-NS3 treated (SGSS05-NS3), collected 20 days after the injection. Data are presented as mean ± SD of three replicates. Statistical significance was calculated using the t test. **E**, **F** NGP NB cells were injected into nude mice. Mice were treated with 0.5 or 1 or 5 mg/kg of SGSS05-NS3 compound intraperitoneally (i.p.) for three weeks. Day 0 indicates the day of cell injection. Day 5 indicates the day of start treatment. Bars show the tumor size average of 6 mice/group ± SEM. Slopes of the growth rate were compared by t test. Images of representative resected xenograft tumors from the indicated mice groups (**E**). Animal weight in the mice groups indicated (**F**). **G**, **H** NGP NB cells were injected into nude mice. Mice were treated with 5 mg/kg of SGSS05-NS3 compound i.p. for three weeks. Day 0 indicates the day of cell injection. Day 5 indicates the day of start treatment. Bars show the tumor size average of 6 mice/group ± SEM. Slopes of the growth rate were compared by t test (**G**). Animal weight in the mice groups indicated (**H**). **I** Images of representative resected xenograft tumors from the indicated mice groups treated as in (**G**). Immunoblot analysis of the indicated total and histone proteins derived from xenograft tumors treated as in (**G**). **J**, **K** Densitometry analysis of p53^K382me1^ protein levels normalized to p53 protein levels (**J**) and of H4^K20me1^ levels normalized to H4 protein levels (**K**) after treatment with SGSS05-NS3 as in (**I**) calculated as relative density units (RDU) using ImageJ software

In order to determine SGSS05-NS3 effects on *in vivo* NB growth, since animal toxicology and bioavailability of SGSS05-NS3 compound did not allow continuous *in vivo* treatment of mice with this SETD8 inhibitor, SY5Y NB cells were exposed ex vivo to 1.5 µM SGSS05-NS3 or DMSO for 24 h *in vitro* and were then injected subcutaneously into nude mice (Fig. 5A). Tumor growth was assessed three times a week. SETD8 pharmacological inhibition with SGSS05-NS3 significantly impaired NB xenograft tumor growth *in vivo* (*p* = 0.0031) (Fig. 5A and Figure S5A). A statistically significant survival advantage for the mice bearing *ex-vivo* SGSS05-NS3-treated SY5Y NB cells compared with control-treated NB cells was revealed by Kaplan–Meier survival curves (*p* = 0.0015) (Fig. 5B). Immunohistochemical analysis of SETD8 and its non-histone target on xenograft tumor tissues indicated that SGSS05-NS3 treatment was able to reduce p53^K382me1^ levels (Fig. 5C insets and Fig. 5D). Next, we performed a preliminary *in vivo* experiment to evaluate the SGSS05-NS3 compound bioavailability, toxicity, and therapeutic feasibility in a NB xenograft model. Using a dose-escalation response curve, we selected 5 mg/kg as an optimal concentration (Fig. 5E-F). We then treated mice injected with NGP NB cells three times a week, for three weeks by administering the selected dose of 5 mg/kg intraperitoneally (i.p.). NGP cells were chosen among the MYCN-amplified NB cells with the highest *in vitro* IC_50_.The continuous *in vivo* treatment with the SGSS05-NS3 compound significantly reduced the NB *in vivo* outgrowth without any evidence of toxicity (Fig. 5G-H). Immunoblot analyses of SETD8 and its histone and non-histone targets on xenograft tumor tissues-derived proteins indicated that SGSS05-NS3 treatment reduced H4^K20me1^ and p53^K382me1^ levels (Fig. 5I-K).

These findings suggest that SGSS05-NS3 compound upon further optimization could potentially be used for *in vivo* preclinical use.

Of note, in MYCN-amplified NB cells (IMR32 and NGP), SETD8 pharmacological inhibition after SGSS05-NS3 treatment led to a dose-dependent reduction of p53^K382me1^ and H4^K20me1^ expression levels (Figures S5B-S5D and Figure S6A-S6B), and to a dose-dependent increase in cell death (Trypan blue positive cells) (Figure S5E and Figure S6C). RNA-seq analysis data revealed that SGSS05-NS3 treatment activates p53 canonical signaling pathway in NGP MYCN-amplified NB cells, with *CDKN1A* (p21), *TNFRSF10B, TP53INP1* and *MDM2* resulting among the top differentially upregulated genes, ranked by statistical significance (FDR < 0.001) (Figure S6D). Consistently, IPA analysis of the differentially expressed genes identified the p53 signaling pathway among the most significantly up-regulated (Figure S6E).

These findings show that SETD8 targeting in NB cells significantly inhibited tumor xenograft growth and prolonged mice survival, paving the way for innovative therapeutic strategies in HR-NBs regardless of MYCN status.

### SETD8 inhibition leads to induction of DNA damage response (DDR) in NB cells

Since transcriptome (bulk RNA-seq) analysis showed that genetic or pharmacological targeting of SETD8 leads to an up-regulation of genes involved in cell apoptosis in response to DNA damage (such as *PIDD1, TNFRSF10B, TP53INP1, FAS*, *GADD45A*, *GADD45B* and *GADD45G*), we evaluated the DNA-damage response (DDR) upon SETD8 inhibition.

**Fig. 6:**
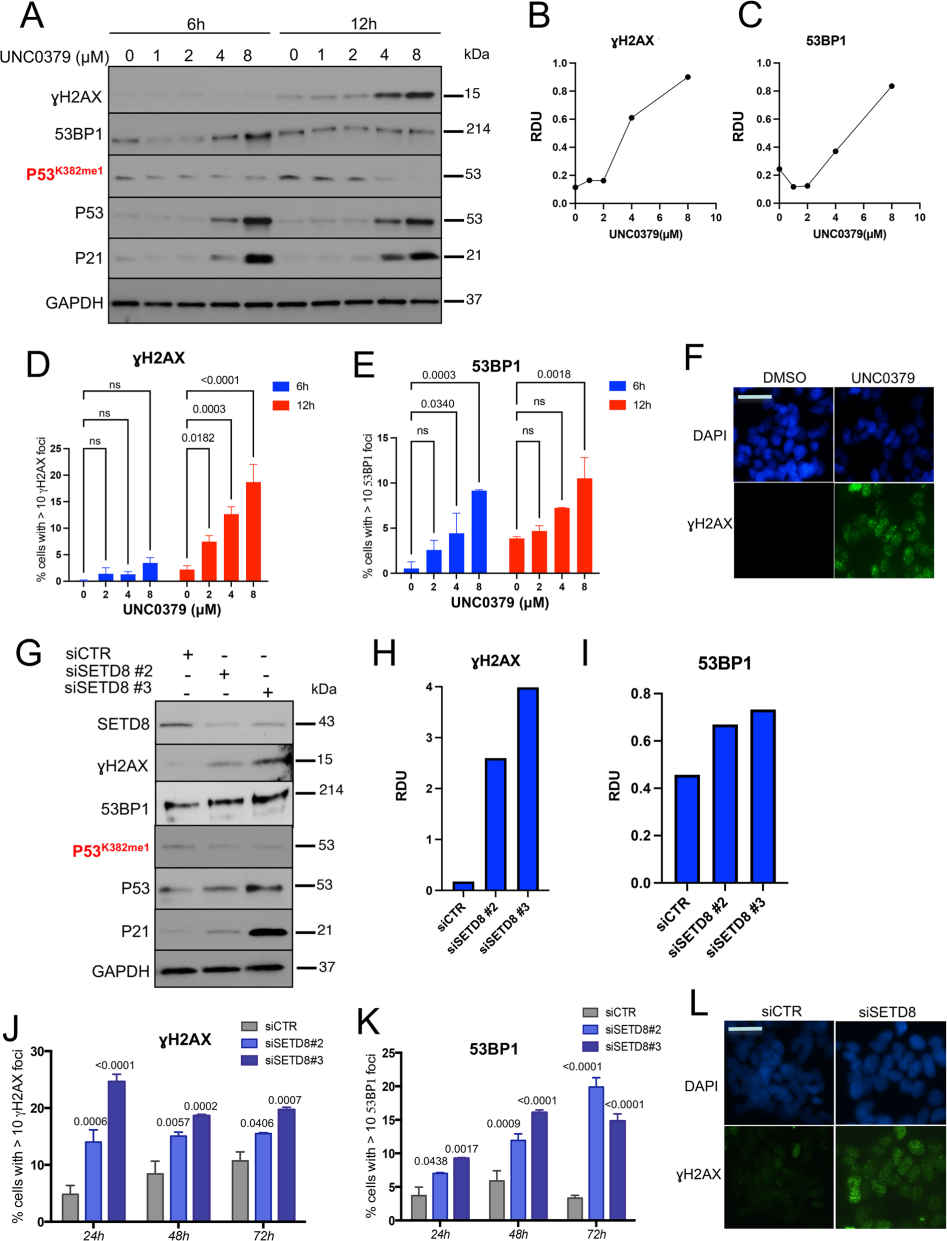
SETD8 pharmacological inhibition leads to induction of DNA damage response (DDR) in NB cells. **A** Immunoblot analysis of the indicated proteins in SY5Y NB cells treated with UNC0379 at the indicated time points and concentrations. **B**, **C** Densitometry analysis of ɣH2AX protein levels normalized to GAPDH protein levels (**B**) and of 53BP1 levels normalized to GAPDH protein levels (**C**) after treatment with UNC0379 at the indicated doses, for 12 and 6 h, respectively, calculated as relative density units (RDU) using ImageJ software. **D**, **E** Percentage of cells with > 10 ɣH2AX (D) or 53BP1 (E) foci in SY5Y NB cells treated with UNC0379 at the indicated time points and concentrations. Data are expressed as mean ± SD of three independent experiments. Statistical significance was calculated using the t test. ns, not significant. **F** Immunofluorescence analysis showing the expression of ɣH2AX (green) after treatment with 4 µM UNC0379 for 12 h in SY5Y NB cells. Nuclei are stained with DAPI (blue). Scale bars, 100 µm. **G** Immunoblot analysis of the indicated proteins in SY5Y NB cells upon genetic inhibition of SETD8 by siRNAs (siSETD8#2 and#3) for 48 h. **H**, **I** Densitometry analysis of ɣH2AX protein levels normalized to GAPDH protein levels (**H**) and of 53BP1 levels normalized to GAPDH protein levels (**I**) upon genetic inhibition of SETD8 by siRNAs (siSETD8#2 and#3) for 48 h, calculated as relative density units (RDU) using ImageJ software. **J**, **K** Percentage of cells with > 10 ɣH2AX (**J**) or 53BP1 (**K**) foci in SY5Y NB cells upon genetic inhibition of SETD8 by siRNAs (siSETD8#2 and#3) at the indicated time points. Data are expressed as mean ± SD of three independent experiments. Statistical significance was calculated using the t test. **L** Immunofluorescence analysis showing the expression of ɣH2AX (green) after SETD8 silencing with siSETD8#3 for 24 h in SY5Y NB cells. Nuclei are stained with DAPI (blue). Scale bars, 100 µm

UNC0379 treatment induces a time- and dose-dependent increase in ɣH2AX or 53BP1 protein levels (Fig. 6A-C) and increased number of NB cells with > 10 ɣH2AX or 53BP1 foci (Fig. 6D-F) as well as SGSS05-NS3 treatment (data not shown). Consistently, SETD8 genetic inhibition by two siRNAs sequences led to an increased DDR in SY5Y NB cells as confirmed by enhanced protein levels of ɣH2AX or 53BP1 (Fig. 6G-I) and increased % of cells presenting more than 10 ɣH2AX or 53BP1 foci (Fig. 6J-L).

These data confirm that targeting SETD8 induces a DNA-damage response and may represent an innovative therapeutic strategy to sensitize NB cells to DDR-inducers drugs.

### Topotecan, a topoisomerase inhibitor, impairs NB cell growth alone and in combination with SETD8 inhibitor, UNC0379 or SGSS05-NS3, enhances the anti-proliferative effects

**Fig. 7:**
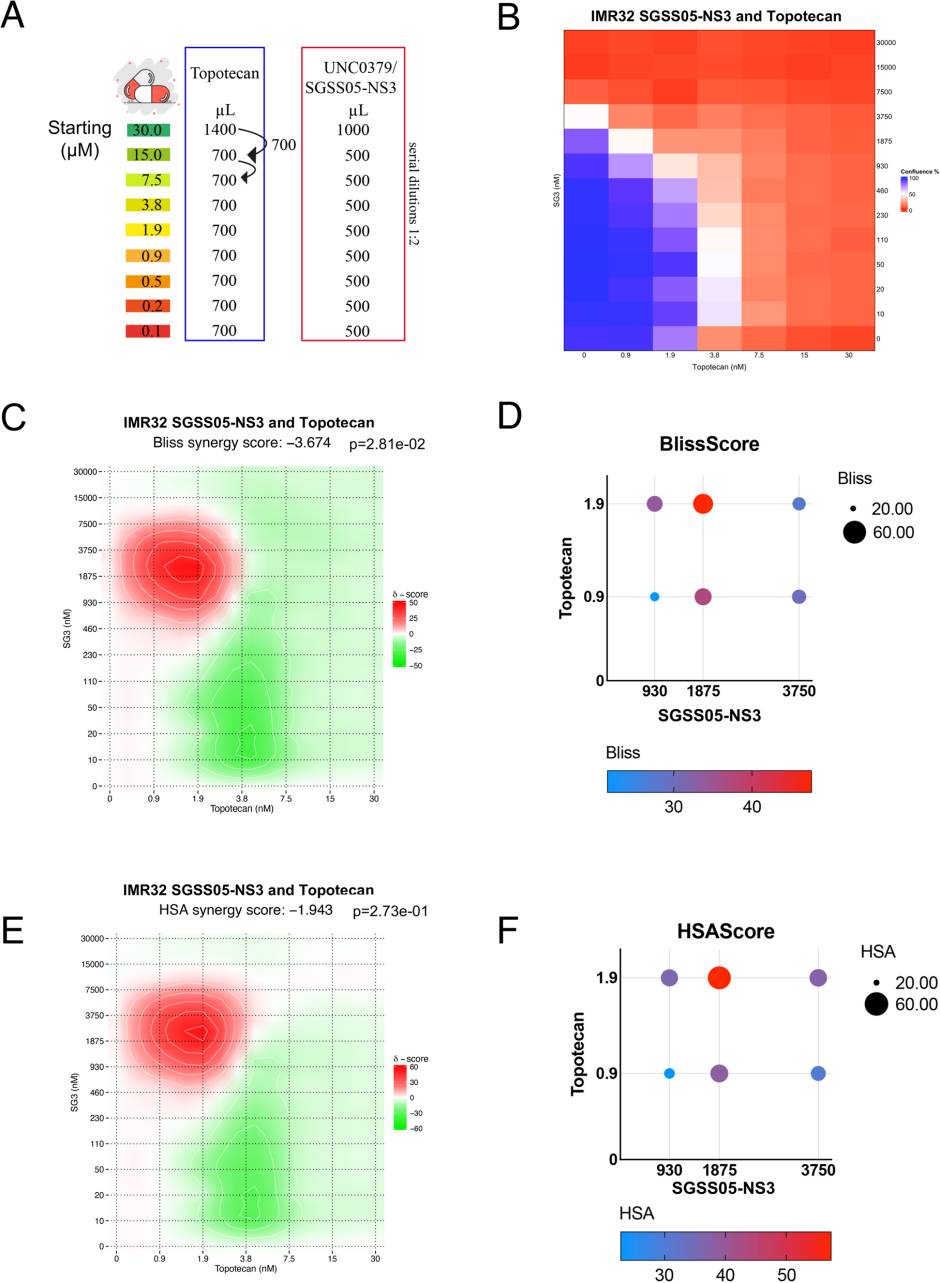
Topotecan, a topoisomerase inhibitor, impairs NB cell growth alone and in combination with SETD8 inhibitor, UNC0379 or SGSS05-NS3, enhances the anti-proliferative effects**. A** Scheme of serial dilutions for synergy score evaluation of Topotecan treatment in combination with SETD8 inhibitor, UNC0379 or SGSS05-NS3, in NB cell lines (*n* = 5). **B** Heatmap shows the percentage of cell confluence upon different concentrations (nM) of Topotecan and SGSS05-NS3, in MYCN-amp NB cells (IMR32) at 96 h. Cell proliferation was measured by Incucyte cell confluence assay. (**C**, **E**) Bliss and HSA synergy scores obtained from the combination of different concentrations (nM) of Topotecan and SGSS05-NS3, in IMR32 NB cells as in (**B**). SynergyFinder online tool is used for bliss or HSA synergistic analysis to evaluate the synergistic effect of the combination treatment in IMR32 NB cells. *P*-values for Synergy scores (Bliss and HSA) were calculated by using t-tests. **D**, **F** Bubble graphs showing Bliss and HSA synergy scores obtained from the combination of the indicated concentrations (nM) of Topotecan and SGSS05-NS3, in IMR32 NB cells. The size and the color of the bubble indicate the relative number of Bliss and HSA synergy scores

In order to assess whether treatment with SETD8 inhibitors (SGSS05-NS3 or UNC0379) could sensitize NB cells to DDR-inducing drugs, we performed a chemical screen evaluating synergy scores, including Bliss and HSA, obtained from the combination of different concentrations of Topotecan (0.1–30 nM), a topoisomerase inhibitor, and SGSS05-NS3 or UNC0379 (0.1–30 µM), in 5 NB cells (2 MYCN-WT, SY5Y and NBLS, and 3 MYCN-amplified, IMR32, SAN and KCNR) (Fig. 7A and Figure S7A). Cell confluence was studied over time up to 7 days.

Firstly, we evaluated the average IC_50_ of Topotecan treatment alone in 5 NB cells, range from 2 to about 8 nM at 96 h (Figure S7A). An illustrative experiment showing Topotecan *in vitro* effects on cell viability in MYCN-amp NB cells, IMR32 and KCNR is depicted in Figures S5B and S5C, respectively. Next, cell confluence measured by IncuCyte, and Bliss and HAS synergy scores obtained from the combination of different concentrations of Topotecan (from 0.1 to 30 nM), and SGSS05-NS3 or UNC0379 (from 0.1 to 30 µM) in the indicated 5 NB cells, were evaluated as the difference between the attended and the observed cell confluency (Fig. 7B-F and Figures S7D and S7E and Table S3). Average synergy scores from −10 to 10 indicate an additive interaction between the two drugs, while greater than 10 indicate a strong synergistic effect. Bliss and HSA synergy scores −3.674 and −1.943, respectively, were obtained in IMR32 MYCN-amp NB cells (*p*-values 2.81e-02 and *p* = 2.73e-01, respectively) (Fig. 7C and E). However, both Bliss and HSA synergy scores indicate that the combination treatment using low dose of SGSS05-NS3 and Topotecan synergistically reduced viable NB cells number with synergy scores greater than 10 (47.57 and 57.33) (Fig. 7D and F, Figure S7F) across a narrow range of doses (SGSS05-NS3 930–3750 nM, Topotecan 0.9–1.9 nM) in IMR32 NB cells. These findings support that even at lower concentration than IC_50_ of SETD8 inhibitor or of Topotecan used alone, these drugs may be used in combination taking advantage from a synergistic effect in terms of reduction of cell confluency and impairment of cell viability. Our data show that Topotecan impairs NB cell growth alone and that the addition of SETD8 inhibitors, SGSS05-NS3 or UNC0379, sensitizes NB cells to Topotecan and enhances the anti-proliferative effects.

**Fig. 8:**
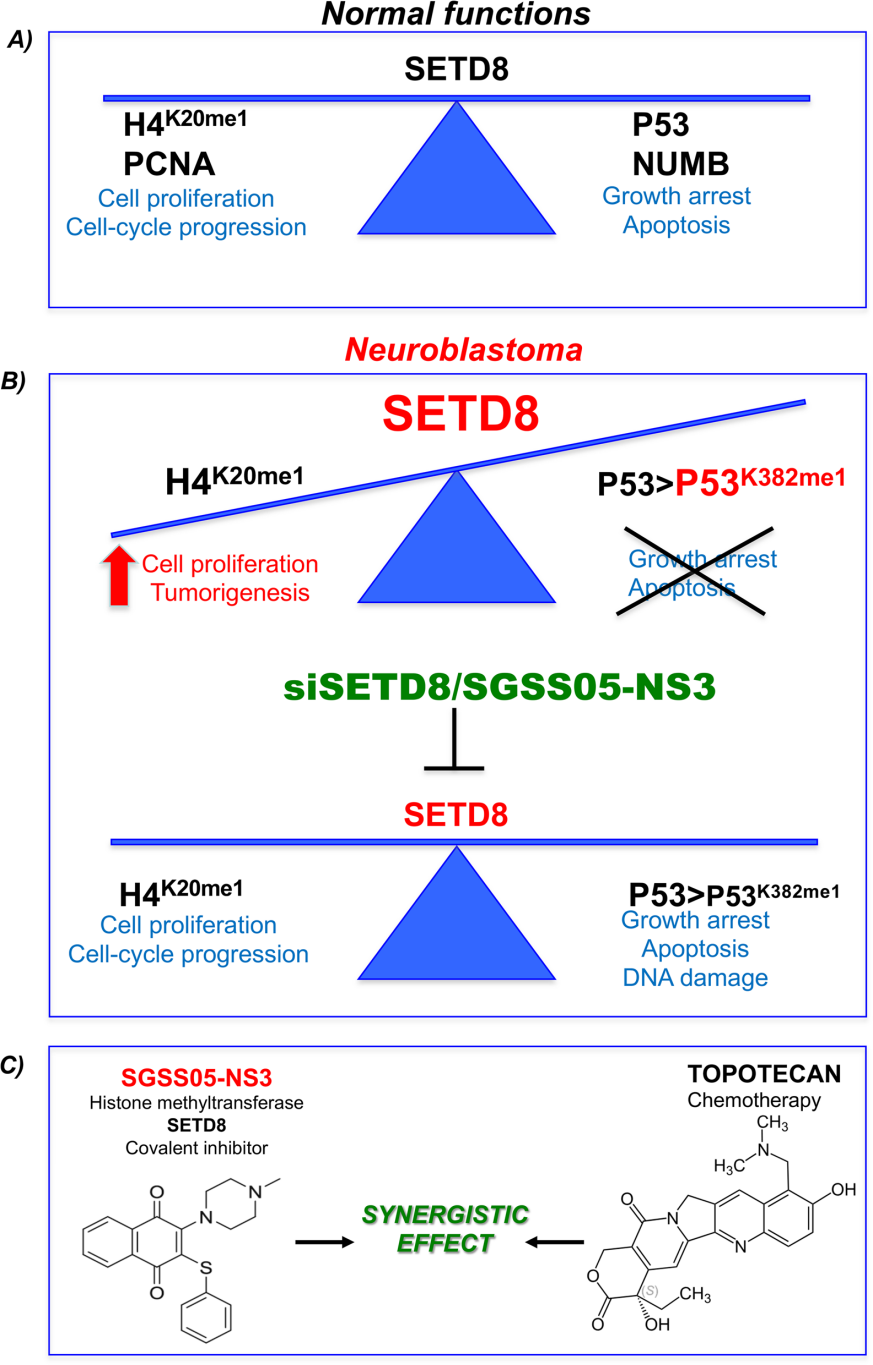
Scheme of SETD8 targeting in NB. **A** In normal conditions SETD8 regulates cell proliferation, cell-cycle progression and growth arrest/apoptosis, through its histone (H4^K20me1^) and non-histone targets (PCNA, p53 and NUMB). **B** In NB tumors, overexpression of SETD8 leads to increased levels of H4^K20me1^ and p53^K382me1^ associated with enhanced cell proliferation and p53 inactivation. SETD8 targeting by genetic (siSETD8) or pharmacological inhibition (SGSS05-NS3 compound, a SETD8 inhibitor) restores p53-mediated cell growth arrest, apoptosis and DNA damage by decreasing p53^K382me1^. **C** SGSS05-NS3 compound, a SETD8 inhibitor, in association with Topotecan enhances the anti-proliferative effects on NB cells

Altogether, our results suggest that SETD8 targeting by genetic (siSETD8) or pharmacological inhibition (SGSS05-NS3 compound or UNC0379), induces DDR and in association with Topotecan enhances the DNA-damage response, restores p53-mediated cell growth arrest and apoptosis by decreasing p53^K382me1^(Fig. 8). This study may open the way to innovative and effective strategies for HR-NBs.

## Discussion

Our study demonstrates that a potent SETD8 inhibitor with enhanced activity and selectivity, SGSS05-NS3 rescues the canonical p53 functions, restores p53-mediated DNA damage response, in pre-clinical xenograft NB models confers a significant survival advantage in MYCN-WT NB. This study provides further evidence for targeting SETD8 as a therapeutic strategy in NB, alone or in combination with Topotecan.

Although we and other groups proposed several therapeutic strategies aimed to sensitizes NB cells to apoptosis and chemotherapy [29–33], identifying more efficacious and less toxic treatments still represents a clinical challenge for pediatric oncologists to manage HR-NB patients.

In the process to develop more specific, potent and cell-active SETD8 inhibitors, in addition to UNC0379, a non-covalent substrate-competitive inhibitor selective for SETD8 over 15 other methyltransferases [23], Jin and Luo groups developed two covalent inhibitors with the desired potency against SETD8, MS453 and SGSS05-NS3 respectively. However, while MS453 showed a low IC50 and selectivity for SETD8 against 28 other methyltransferases suffered from poor membrane permeability which limited the *in vivo* efficacy [34]. Thus, we focused on SGSS05-NS3 as a covalent [35] cell-permeable, SETD8 inhibitor with higher *in vitro* therapeutic index (IVTI) and less toxicity on normal cells compared to UNC0379.

SGSS05-NS3 compound has been developed and patented by Minki Luo and Gil Blum at Sloan-Kettering Institute (Luo M, Sanchez GI, Blum GJ, Yang L (Sloan-Kettering Institute for Cancer Research, USA), 2015, WO2015172076A1(WO2015US29977 20,150,508), 152 pp). It has been included in a series of naphthoquinone derivatives, the more active of them (namely, compounds SGSS05-N, SGSS05-NS and SPECS-21) inhibited SETD8 with IC_50_ values ≤ 5 μM, although the compounds also inhibited other KMTs (SETD2, SETDB1, GLP, G9a, SMYD2, SMYD3, MLL1, and SETD7) and PRMTs (PRMT1, PRMT3, CARM1, PRMT8) in the low micromolar range.

In our study, SGSS05-NS3 compound had a highly significant *In Vitro* Therapeutic Index (IVTI) across several NB cell lines and non-transformed cells, with enhanced activity at reactivating p53 functions and increasing p53 total protein levels in both MYCN-WT and amplified NB cells, supporting the use of this drug in a broad range of HR-NBs. Compared to the other two SETD8 inhibitors, UNC0379, a substrate-competitive SETD8 inhibitor with preferential selectivity for SETD8 compared with 15 other methyltransferases [23, 36], and SPECS-21 (Luo M, Sanchez GI, Blum GJ, Yang L (Sloan-Kettering Institute for Cancer Research, USA), 2015, WO2015172076A1(WO2015US29977 20,150,508), 152 pp), the SGSS05-NS3 compound showed a significantly lower *in vitro* IC_50_ and higher IVTI.

Of note, SGSS05-NS3 *ex-vivo* treatment significantly impaired tumor growth and prolonged murine survival in preclinical *in vivo* models of NB. In preclinical NB models SGSS05-NS3 treatment was firstly performed *ex-vivo*, since animal toxicology and bioavailability of SGSS05-NS3 compound did not allow continuous *in vivo* treatment of mice with this SETD8 inhibitor. This limitation based solely on the limited data available regarding SGSS05-NS3’s toxicology and bioavailability, could potentially be overcome, as it has been the case with other SETD8 inhibitors such as UNC0379 [37]. Secondly, we performed an *in vivo* dose-escalation response curve to select an optimal concentration of SGSS05-NS3 compound. The continuous *in vivo* SGSS05-NS3 treatment significantly reduced *in vivo* NB outgrowth without any sign of toxicity. Although the *in vivo* results are promising, it is worth pointing out that unlike SGSS05-NS3, another covalent SETD8 inhibitor, MS453, was reported. However, due to poor bioavailability, no further studies have been carried out. As the “non-covalent” affinity of SETD8 inhibitors like UNC0379 is in general low, we believe that the information provided by our current data with SGSS05-NS3 will help in identifying new compounds potentially useful for future *in vivo* experiments.

Notably transcriptome (bulk RNA-seq) analysis pointed out that SGSS05-NS3 treatment compared with UNC0379 or with the SETD8 genetic inhibition [11], specifically activates genes involved in cell apoptosis in response to DNA damage such as *GADD45A, GADD45B* and *GADD45G,* along with *PIDD1, TNFRSF10B, TP53INP1* and *FAS,* supporting an increased activity and specificity of SGSS05-NS3 in restoring p53 functions and increasing p53 protein levels.

In our study, we evaluated DNA-damage response (DDR) upon SETD8 pharmacological and genetic inhibition in NB cells. Although SETD8’s role in DNA damage repair has been studied, along with its fine regulation during cell-cycle and mitotic progression by ubiquitin-dependent proteasomal degradation [13, 21, 22, 38, 39], its function in NB cells has not been extensively clarified.

Here, we found that SETD8 inhibition led to DDR induction in NB cells. Specifically pharmacological and genetic inhibition of SETD8 in NB cells induced a time- and dose-dependent increase in ɣH2AX or 53BP1 protein levels and an increased number of NB cells with > ɣH2AX or 53BP1 10 foci. Our results support that SETD8 inhibition will sensitize cancer cells to checkpoint inhibitors or standard chemotherapy by inducing DNA damage in NB. Moreover, we found that in combination with DNA-damage response inducers, such as Topotecan, SGSS05-NS3 showed synergistic effects in reducing cell proliferation of NB cells. This study may pave the way to the use of a combinatorial treatment which may overcome therapy resistance [40–43].

In our study, SETD8 targeting increases 53BP1 and ɣH2AX recruitment to DNA damage sites, likely leading to an increased repair through non-homologous end joining (NHEJ). This phenomenon may be associated with an increased mutational burden in NB cells which combined with blockade of topoisomerases by Topotecan, may further increase the mutational burden, supporting the hypothesis that SGSS05-NS3 or UNC0379 treatment in combination with Topotecan may potentially have a synthetic lethality effect [44–47].

Altogether, our findings shed new lights on the role of SETD8 in NB as a master regulator of proliferative plasticity and DNA damage repair and response, paving the way for innovative therapeutic combinatorial strategies which take advantage of the synergistic effect of the SGSS05-NS3 compound in combination with Topotecan.

## Conclusions

This study aims to highlight the efficacy of SGSS05-NS3 as a covalent and cell-permeable inhibitor targeting SETD8. SGSS05-NS3 exhibits a significantly lower IC_50_ value and higher *in vitro* therapeutic index (IVTI) compared with the previous established substrate-competitive SETD8 inhibitor, UNC0379. Of note, SGSS05-NS3 is more selective for NB cells over non-tumor control cells, with a better safety profile and lower toxicity to normal cells, representing a significant advancement in the field of covalent SETD8 inhibitors, as the “non-covalent” affinity of SETD8 inhibitors like UNC0379 is in general low. Furthermore, SGSS05-NS3 rescues p53 canonical functions specifically activating genes involved in growth arrest, apoptosis and DNA damage response (DDR) such as *CDKN1A (p21), GADD45A, GADD45B, GADD45G, FAS* and *PMAIP (Noxa*), showing strong synergy in combination with DDR-inducers chemotherapeutic drugs such as Topotecan in NB cells. Notably, in preclinical NB xenograft models, SGSS05-NS3 reduces tumor growth, suggesting its potential as a therapeutic agent.

## Supplementary Information

Supplementary material 1.

## Data Availability

The RNA-seq data generated in this study are publicly available in Gene Expression Omnibus (GEO) at GSE270115, and within the article and its supplementary data files. All the reagents and data generated in this study are available upon request from the corresponding author.

## Abbreviations

DEGs: Differential expression genes
FDR: False discovery rate
GEO: Gene expression omnibus
GSEA: Gene set enrichment analysis
H&E: Hematoxylin and eosin
HR-NB: High-risk neuroblastoma
IF: Immunofluorescence
IHC: Immunohistochemistry
IPA: Ingenuity pathway analysis
IVTI: *In vitro* therapeutic index
KMTs: Lysine methyltransferases
NHEJ: Non-homologous end joining
PRMTs: Protein arginine methyltransferase
RNA-seq: RNA sequencing
