# Supplementary material for: SGSS05-NS3, a covalent SETD8 inhibitor that activates p53 pathway in neuroblastoma
Source: J Exp Clin Cancer Res. 2025 Dec 19;45:5. doi: 10.1186/s13046-025-03565-7 (PMC12766961; doi:10.1186/s13046-025-03565-7)
Supplement: Supplementary file 1 — Supplementary material 1. [file 13046_2025_3565_MOESM1_ESM.pdf]

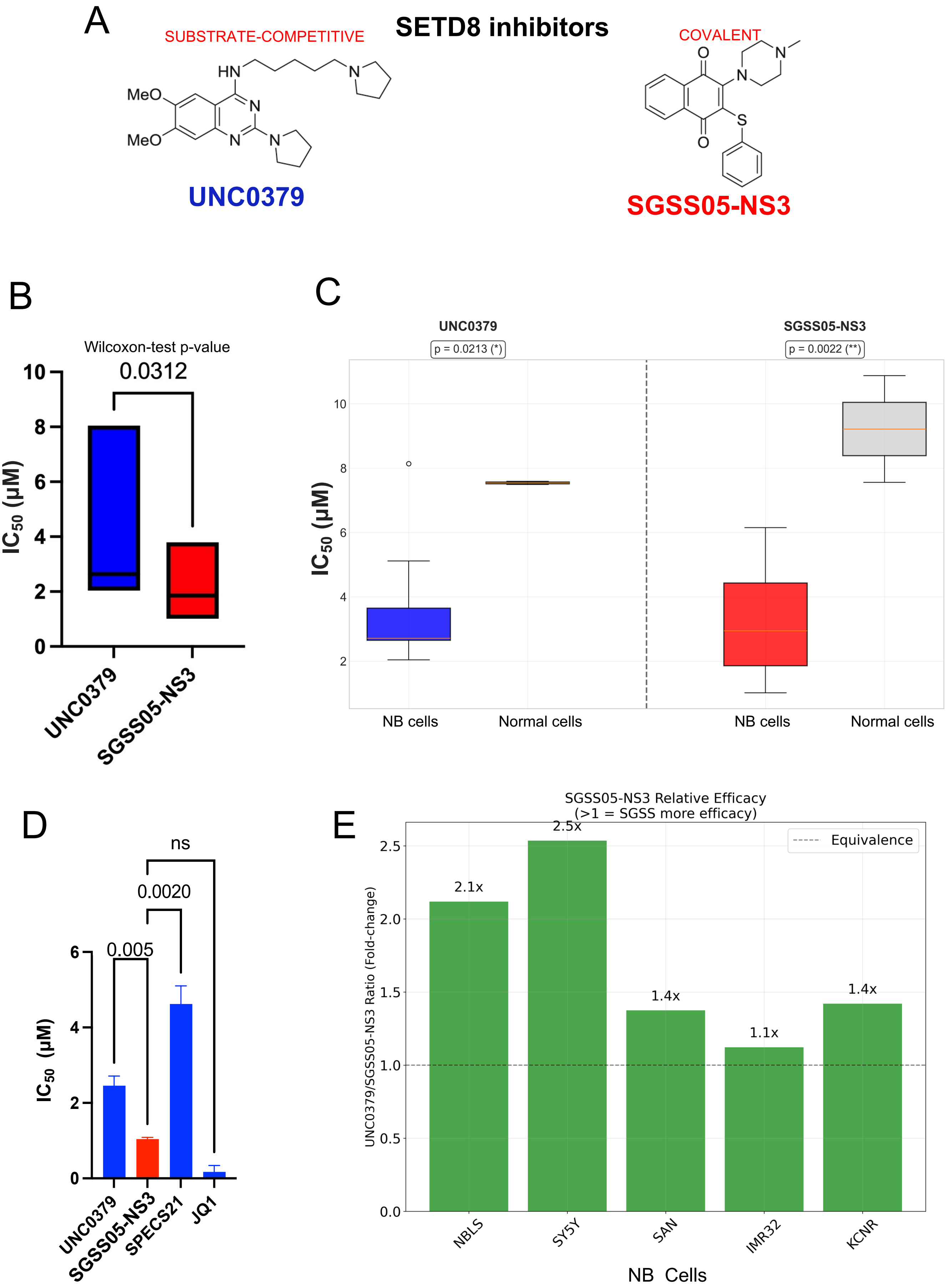
A
SETD8 inhibitors
SUBSTRATE-COMPETITIVE
UNC0379
COVALENT
SGSS05-NS3
B
Wilcoxon-test p-value
0.0312
IC50 (μM)
UNC0379
SGSS05-NS3
C
UNC0379
p = 0.0213 (*)
SGSS05-NS3
p = 0.0022 (**)
IC50 (μM)
NB cells
Normal cells
NB cells
Normal cells
D
ns
0.0020
0.005
IC50 (μM)
UNC0379
SGSS05-NS3
SPECS21
JQ1
E
SGSS05-NS3 Relative Efficacy
(>1 = SGSS more efficacy)
Equivalence
2.1x
2.5x
1.4x
1.1x
1.4x
UNC0379/SGSS05-NS3 Ratio (Fold-change)
NBLS
SY5Y
SAN
IMR32
KCNR
NB Cells

Figure S1

F

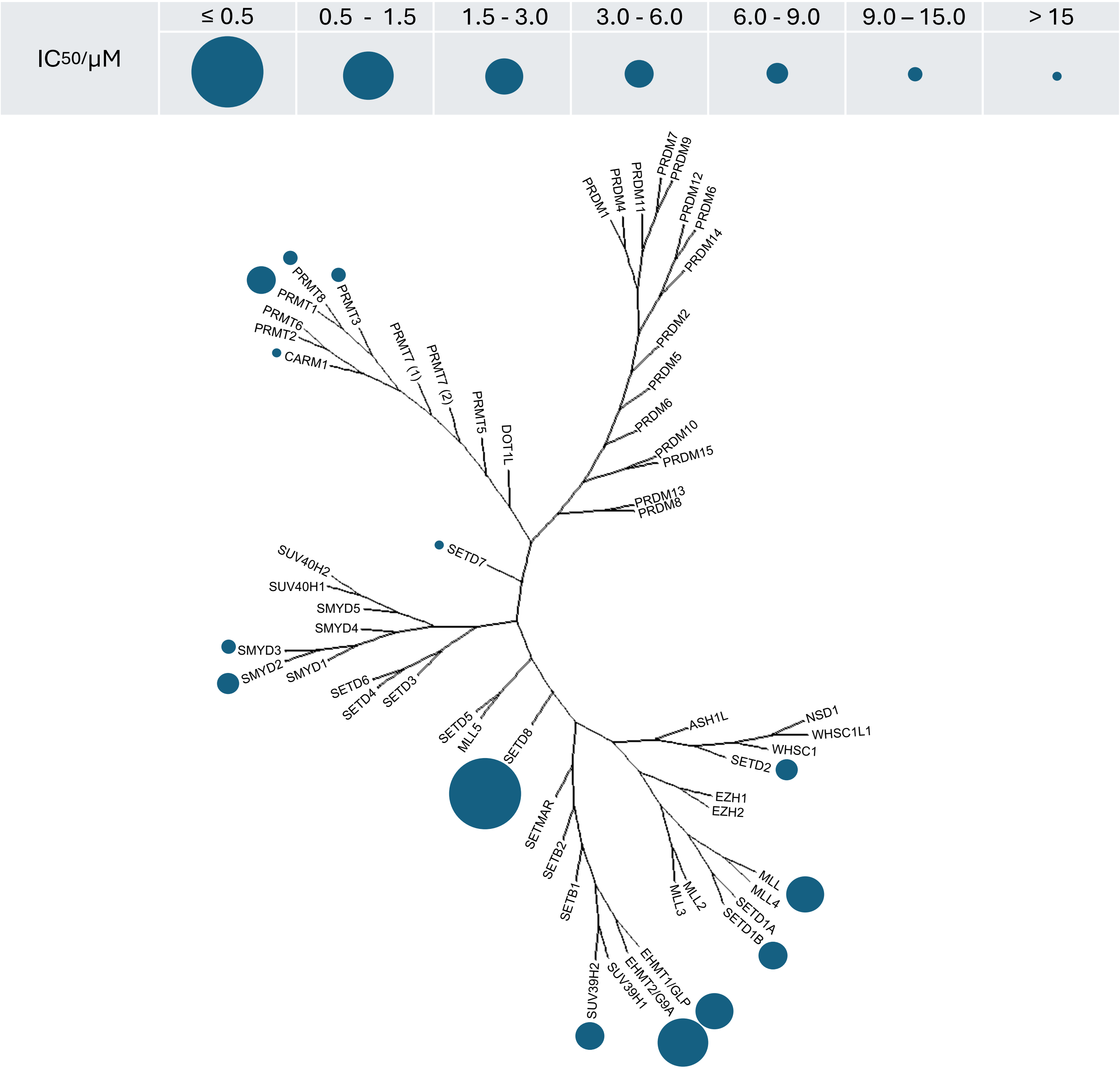

IC50/µM	SETD8	G9a	GLP1	MLL1	PRMT1	SETDB1	SUV39H2	SETD2	SMYD2	PRMT8	SMYD3	PRMT3	Set7/9	CARM1
Gene Name	*KMT5A*	*EHMT2*	*EHMT1*	*KMT2A*	*PRMT1*	*SETDB1*	*SUV39H2*	*SETD2*	*SMYD2*	*PRMT8*	*SMYD3*	*PRMT3*	*SETD7*	*CARM1*
**SGSS05-NS3**	0,5	1,4	1,6	1,6	3,2	5,4	5,9	6	8,8	10,6	13,5	13,9	>50	>50

Figure S1

Figure S1, related to Figure 1

SGSS05-NS3 is a covalent SETD8 inhibitor effective in both MYCN-WT and amplified NB cells

A) Chemical structure of the two compounds SETD8 inhibitors, UNC0379 and SGSS05-NS3.

B) IC_{50} (µM) at 96 hours were compared between the two SETD8 inhibitors, UNC0379 (blue) and SGSS05-NS3 (red) in NB cell lines indicated in Table S1 using the Wilcoxon test (p-value = 0.0312).

C) IC_{50} (µM) at 96 hours were compared between the two SETD8 inhibitors UNC0379 and SGSS05-NS3 in NB cell lines versus normal cell lines indicated in Table S1 with an in vitro therapeutic index (IVTI) (2.8 vs. 2.0). Statistical significance was assessed using Student's t-test (p-value=0.0213 UNC0379; p-value=0.0022 SGSS05-NS3).

D) Average IC_{50} (µM) values of four epigenetic compounds, SETD8 inhibitors (UNC0379, SGSS05-NS3 and SPECS21) and BET inhibitor (JQ1), in SY5Y MYCN-WT NB cells at 96 hours. Data are presented as mean ± SD of three independent experiments. Statistical significance was calculated using the t test. ns, not significant.

E) UNC0379/SGSS05-NS3 Ratio (Fold-change) based on relative IC_{50} values in the indicated NB cell lines at 96 hours. Values >1 indicates a higher SGSS05-NS3 efficacy.

F) Selectivity/Specificity of SGSS05-NS3 across a panel of protein lysine methyltransferases (PKMTs). IC_{50} values for SGSS05-NS3 were determined against fourteen phylogenetically related PKMTs (numerical IC_{50} values are reported in the table beneath the tree). Circle diameter is inversely proportional to the IC50 value; larger circles indicate greater inhibitory potency

Table S1, related to Figure 1

Average IC_{50} (µM) of the two epigenetic compounds SETD8 inhibitors, UNC0379 and SGSS05-NS3, in the indicated MYCN-WT (blue color) and MYCN-amplified (red color) NB cells compared with control cells at 72 hours and 96 hours. In vitro therapeutic index (IVTI) and p-values are shown. Statistical significance was calculated using the t test. Provided as an Excel file.

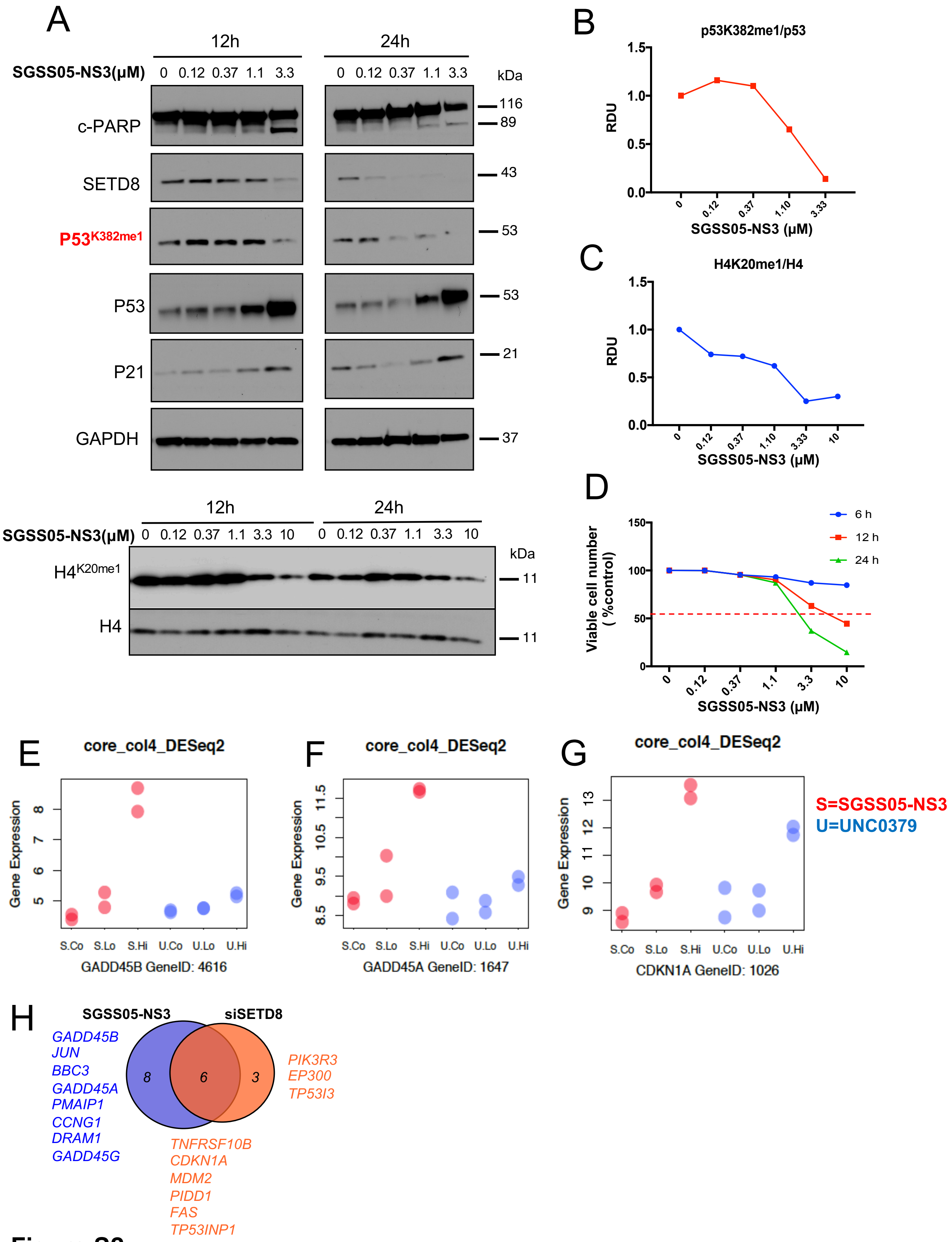
A
12h
24h
SGSS05-NS3(μM)
0 0.12 0.37 1.1 3.3
kDa
c-PARP
116
89
SETD8
43
P53K382me1
53
P53
53
P21
21
GAPDH
37
SGSS05-NS3(μM) 0 0.12 0.37 1.1 3.3 10
H4K20me1
11
H4
11
B
p53K382me1/p53
RDU
SGSS05-NS3 (μM)
C
H4K20me1/H4
RDU
SGSS05-NS3 (μM)
D
Viable cell number (%control)
6 h
12 h
24 h
SGSS05-NS3 (μM)
E
core_col4_DESeq2
Gene Expression
S.Co S.Lo S.Hi U.Co U.Lo U.Hi
GADD45B GeneID: 4616
F
core_col4_DESeq2
Gene Expression
GADD45A GeneID: 1647
G
core_col4_DESeq2
Gene Expression
CDKN1A GeneID: 1026
S=SGSS05-NS3
U=UNC0379
H
SGSS05-NS3
siSETD8
GADD45B
JUN
BBC3
GADD45A
PMAIP1
CCNG1
DRAM1
GADD45G
8
6
3
PIK3R3
EP300
TP53I3
TNFRSF10B
CDKN1A
MDM2
PIDD1
FAS
TP53INP1

Figure S2

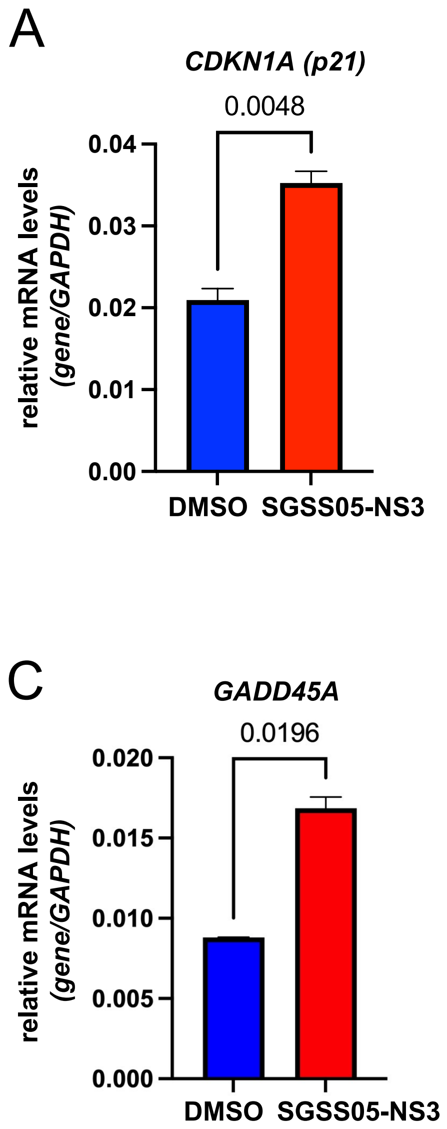
A
CDKN1A (p21)
0.0048
relative mRNA levels
(gene/GAPDH)
0.04
0.03
0.02
0.01
0.00
DMSO
SGSS05-NS3
B
FAS
0.0402
0.0008
0.0006
0.0004
0.0002
0.0000
C
GADD45A
0.0196
0.020
0.015
0.010
0.005
0.000
D
GADD45B
0.0201
0.0004
0.0003
0.0002
0.0001
0.0000

Figure S3

Figure S2, related to Figure 2

SGSS05-NS3 compound specifically activates p53 target genes involved in growth arrest, apoptosis and DNA-damage response, such as GADD45A, GADD45B and GADD45G

A) Immunoblot analysis of the indicated total and histone proteins in SY5Y MYCN-WT NB cells treated with SGSS05-NS3 at the indicated time points and concentrations (serial dilutions 1:3).

B, C) Densitometry analysis of $p53^{K382me1}$ protein levels normalized to p53 protein levels (B) and of $H4^{K20me1}$ levels normalized to H4 protein levels (C) after treatment with the SETD8 inhibitor, SGSS05-NS3, for 12 hours calculated as relative density units (RDU) using ImageJ software.

D) Viable cell number in SY5Y NB cells treated with the indicated concentrations of SGSS05-NS3 at the indicated time points. Data are presented as % over control ± SD of three independent experiments.

E, F, G) Gene expression values from RNA-seq data of *GADD45B, GADD45A* and *CDKN1A* (p21) genes in SY5Y NB cells treated with low and high concentrations of UNC0379 (2µM vs 4µM) or SGSS05-NS3 (1,5µM vs 3µM) compounds for 12 hours (S=SG3, U= UNC0379, Co= control, Lo=Low, Hi=High).

H) Venn diagram of common (n=6) and exclusive upregulated p53 target genes upon 3 µM SGSS05-NS3 treatment for 12 hours and after SETD8 silencing by siRNA for 36 hours, respectively, in SY5Y MYCN-WT NB cells.

Figure S3, related to Figure 2

A, B, C, D) Relative mRNA expression levels of *CDKN1A* (p21), *FAS, GADD45A* and *GADD45B* in SY5Y treated with SGSS05-NS3 (1,5 µM) for 12 hr. Data are presented as mean ± SD of three independent experiments. Statistical significance was calculated using the t test.

Table S2, related to Figure 2

P53 downstream pathway gene list enriched in SETD8 pharmacological inhibition by SGSS05-NS3 compound. Provided as an Excel file.

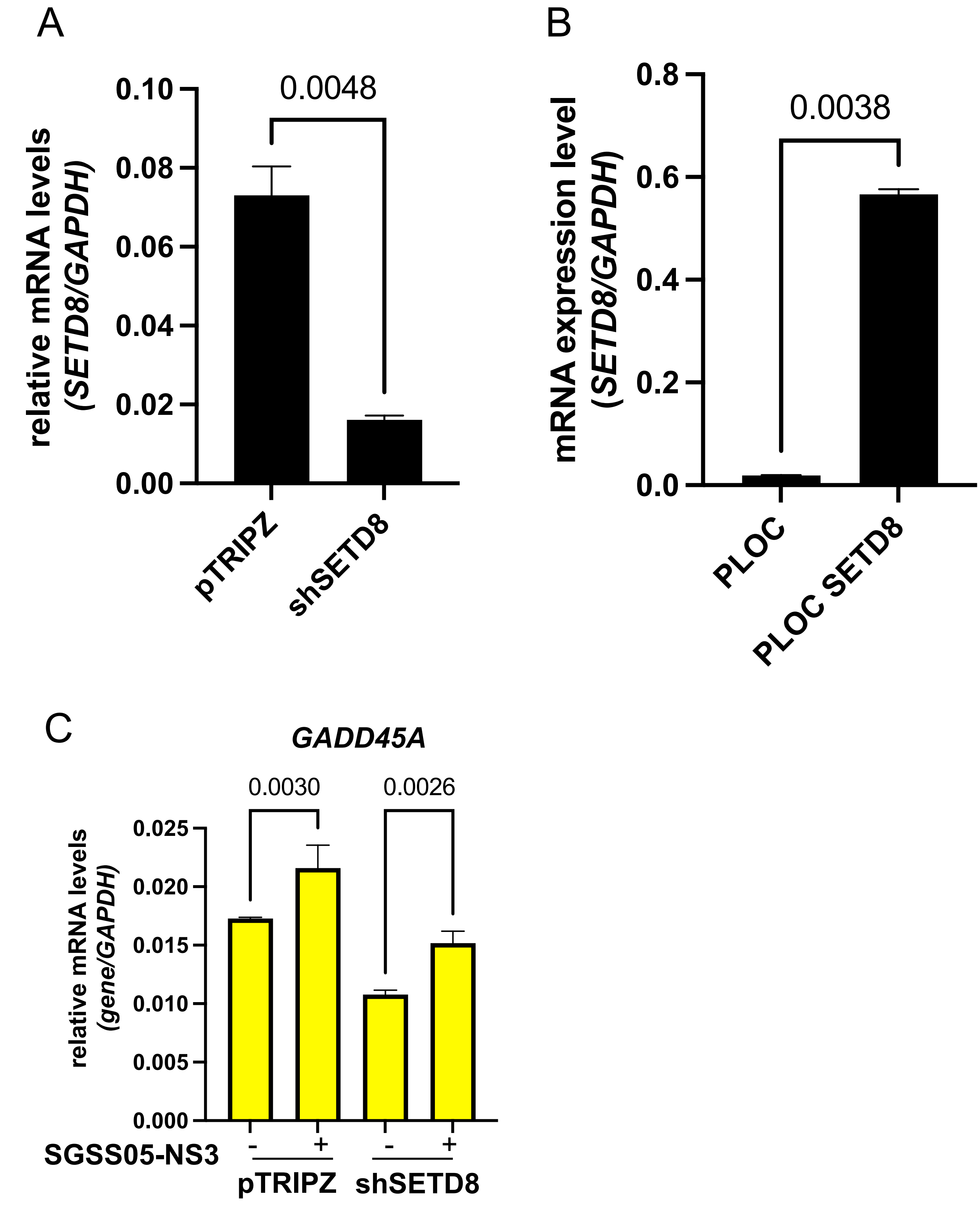
A
0.0048
0.10
0.08
0.06
0.04
0.02
0.00
relative mRNA levels
(SETD8/GAPDH)
pTRIPZ
shSETD8
B
0.0038
0.8
0.6
0.4
0.2
0.0
mRNA expression level
(SETD8/GAPDH)
PLOC
PLOC SETD8
C
GADD45A
0.0030
0.0026
0.025
0.020
0.015
0.010
0.005
0.000
relative mRNA levels
(gene/GAPDH)
SGSS05-NS3
-
+
-
+
pTRIPZ
shSETD8

Figure S4

Figure S4, related to Figure 4

SGSS05-NS3 compound activates the p53 pathway via SETD8 inhibition in NB cells

A) Relative mRNA expression levels of *KMT5A* (SETD8) in SY5Y NB cells upon transfection with pTRIPZ (empty vector) or shSETD8 for 24 hr. Bars show the mean ± SD of three replicates.

B) Relative mRNA expression levels of *KMT5A* (SETD8) in SK-N-SH NB cells upon transfection with PLOC (empty vector) or PLOC SETD8 (SETD8) for 24 hr. Bars show the mean ± SD of three replicates.

C) Relative mRNA expression levels of *GADD45A* in SY5Y NB cells upon transfection with pTRIPZ (empty vector) or shSETD8, treated with SGSS05-NS3 2μM for 24 hr. Bars show the mean ± SD of three replicates.

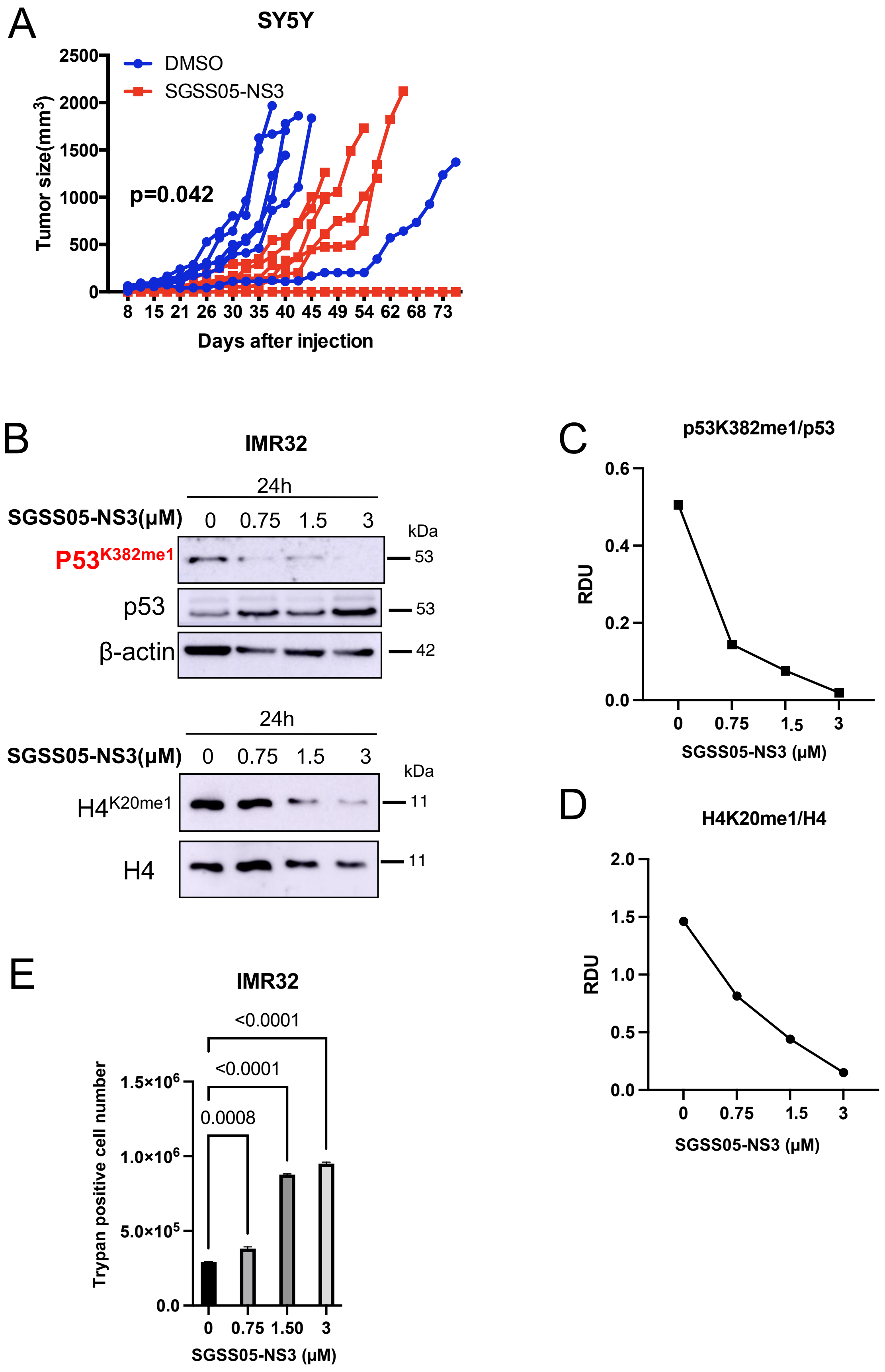
A
SY5Y
DMSO
SGSS05-NS3
Tumor size(mm3)
2500
2000
1500
1000
500
0
p=0.042
8 15 21 26 30 35 40 45 49 54 62 68 73
Days after injection
B
IMR32
24h
SGSS05-NS3(μM)
0 0.75 1.5 3
kDa
P53K382me1
p53
β-actin
53
53
42
24h
SGSS05-NS3(μM)
0 0.75 1.5 3
kDa
H4K20me1
H4
11
11
C
p53K382me1/p53
RDU
0.6
0.4
0.2
0.0
0 0.75 1.5 3
SGSS05-NS3 (μM)
D
H4K20me1/H4
RDU
2.0
1.5
1.0
0.5
0.0
0 0.75 1.5 3
SGSS05-NS3 (μM)
E
IMR32
<0.0001
<0.0001
0.0008
Trypan positive cell number
1.5×10^6
1.0×10^6
5.0×10^5
0.0
0 0.75 1.50 3
SGSS05-NS3 (μM)

Figure S5

Figure S5, related to Figure 5

SETD8 pharmacological inhibition upon SGSS05-NS3 treatment reduces p53^{K382me1} and increases cell death in IMR32 MYCN-amplified NB cells

A) SY5Y cells were treated ex-vivo with 1,5 μM SGSS05-NS3 for 24 hours and then injected into nude mice. Day 0 indicates the day of cell injection. Tumor size of each one of the 15 mice/group derived from the two groups (untreated and ex-vivo SGSS05-NS3 treated) is shown.

B) Immunoblot analysis of the indicated total and histone proteins in IMR32 MYCN-amp NB cells treated with SGSS05-NS3 at the indicated concentrations for 24 hours.

C, D) Densitometry analysis of p53^{K382me1} protein levels normalized to p53 protein levels (C) and of H4^{K20me1} levels normalized to H4 protein levels (D) after treatment with the SETD8 inhibitor, SGSS05-NS3, for 24 hours calculated as relative density units (RDU) using ImageJ software.

E) Trypan positive cell number in SY5Y NB cells treated with the indicated concentrations of SGSS05-NS3 for 24 hours. Data are presented as mean ± SD of three independent experiments. Statistical significance was calculated using the t test.

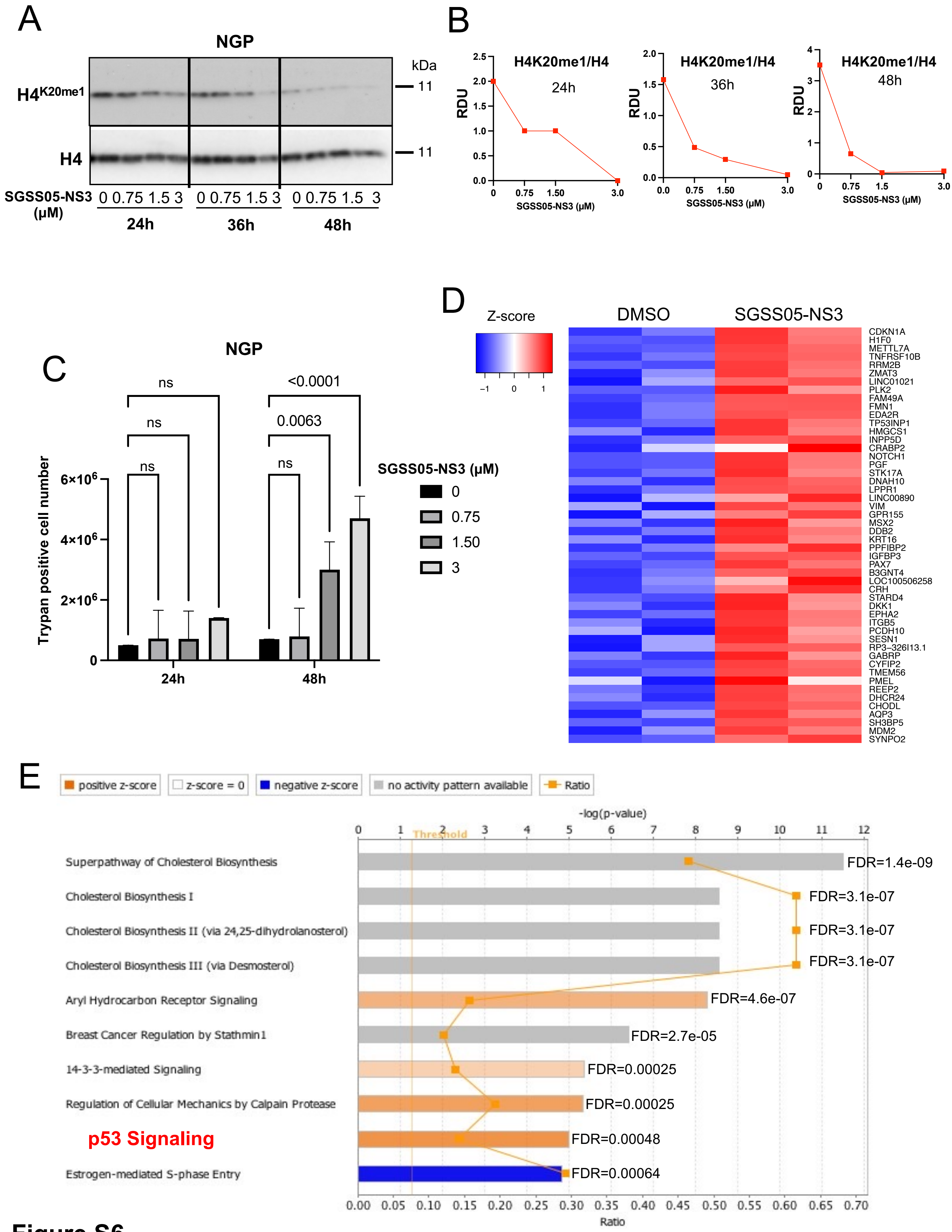
A
NGP
H4K20me1
H4
kDa
11
11
SGSS05-NS3 (μM)
0 0.75 1.5 3
24h
36h
48h
B
H4K20me1/H4
24h
36h
48h
RDU
SGSS05-NS3 (μM)
C
NGP
ns
<0.0001
0.0063
Trypan positive cell number
SGSS05-NS3 (μM)
0
0.75
1.50
3
24h
48h
D
Z-score
DMSO
SGSS05-NS3
CDKN1A
H1F0
METTL7A
TNFRSF10B
RRM2B
ZMAT3
LINC01021
PLK2
FAM49A
FMN1
EDA2R
TP53INP1
HMGCS1
INPP5D
CRABP2
NOTCH1
PGF
STK17A
DNAH10
LPPR1
LINC00890
VIM
GPR155
MSX2
DDB2
KRT16
PPFIBP2
IGFBP3
PAX7
B3GNT4
LOC100506258
CRH
STARD4
DKK1
EPHA2
ITGB5
PCDH10
SESN1
RP3-326I13.1
GABRP
CYFIP2
TMEM56
PMEL
REEP2
DHCR24
CHODL
AQP3
SH3BP5
MDM2
SYNPO2
E
positive z-score
z-score = 0
negative z-score
no activity pattern available
Ratio
-log(p-value)
Threshold
Superpathway of Cholesterol Biosynthesis
Cholesterol Biosynthesis I
Cholesterol Biosynthesis II (via 24,25-dihydrolanosterol)
Cholesterol Biosynthesis III (via Desmosterol)
Aryl Hydrocarbon Receptor Signaling
Breast Cancer Regulation by Stathmin1
14-3-3-mediated Signaling
Regulation of Cellular Mechanics by Calpain Protease
p53 Signaling
Estrogen-mediated S-phase Entry
FDR=1.4e-09
FDR=3.1e-07
FDR=3.1e-07
FDR=3.1e-07
FDR=4.6e-07
FDR=2.7e-05
FDR=0.00025
FDR=0.00025
FDR=0.00048
FDR=0.00064
Ratio

Figure S6

Figure S6, related to Figure 5

RNA-seq data show that SGSS05-NS3 treatment activates p53 canonical signaling pathway in NGP MYCN-amplified NB cells

A) Immunoblot analysis of the indicated histone proteins in NGP MYCN-amp NB cells treated with SGSS05-NS3 at the indicated time points and concentrations.

B) Densitometry analysis of $H4^{K20me1}$ levels normalized to H4 protein levels after treatment with the SETD8 inhibitor, SGSS05-NS3, for 24, 36 and 48 hours calculated as relative density units (RDU) using ImageJ software.

C) Trypan blue positive cell number in NGP MYCN-amp NB cells treated with the indicated concentrations of SGSS05-NS3 at the indicated time points. Data are expressed as mean ± SD of three independent experiments. Statistical significance was calculated using the t test. ns, not significant.

D) Heatmap of the top 50 up-regulated genes in NGP MYCN-amp NB cells ranked by statistical significance following 12 hours of treatment with 3 μM (IC80) SGSS05-NS3. Data are presented as normalized expression values of two biological replicates based on edgeR software analysis and FDR <0.001. The color key represents the normalized expression values: blue (low) to red (high).

E) The top ten differentially expressed canonical pathways after SETD8 pharmacological inhibition by SGSS05-NS3 treatment in NGP cells treated as in (C), defined by Ingenuity Pathway Analysis (IPA) based on edgeR software analysis and FDR <0.001. Pathways related to p53 signaling are among the top differentially expressed pathways.

A

Topotecan alone IC50	12 Hours	24 Hours	48 Hours	72 Hours	96 Hours	120 Hours
KCNR	4.197	4.863	6.954	6.151	7.663	8.242
SAN	7.002	0.9342	10.55	4.388	3.942	3.779
SY5Y	4.734	n.d.	n.d.	n.d.	7.745	3.791
IMR32	2.444	4.365	3.489	2.501	2.533	2.748
NBLS	n.d.	18.55	6.007	3.368	2.365	2.303

B

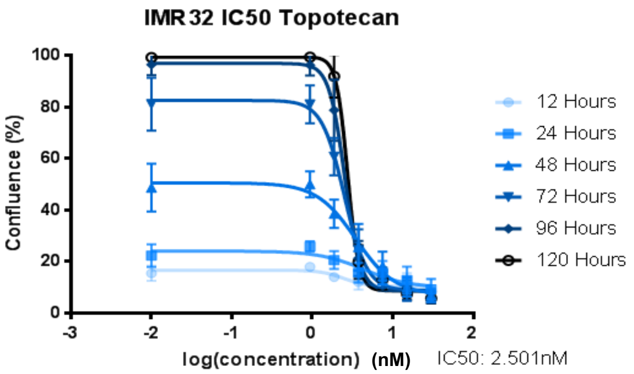

C

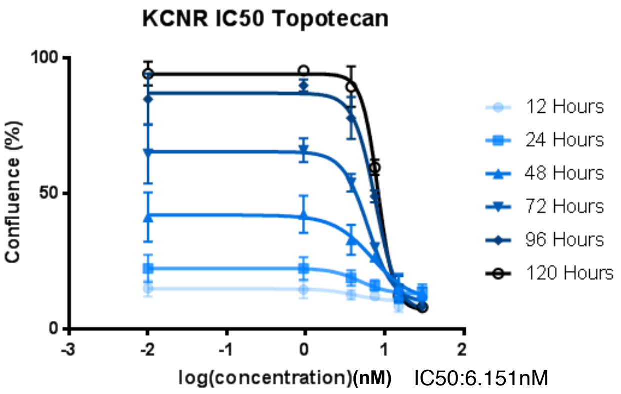

D

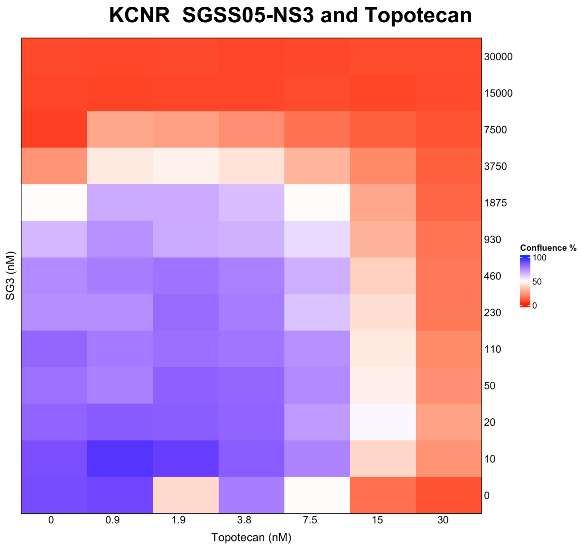

E

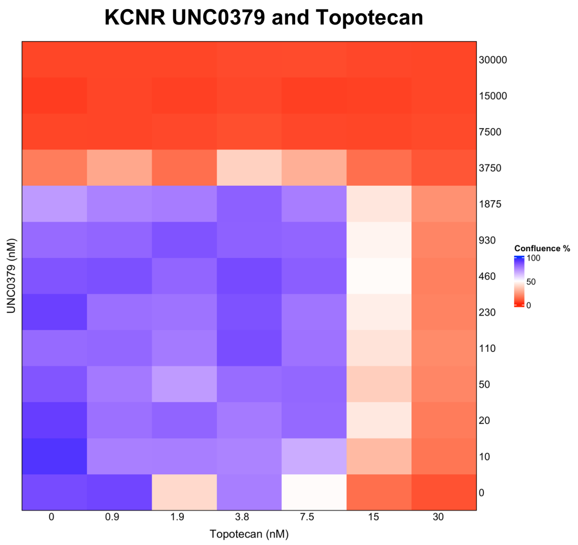

Figure S7

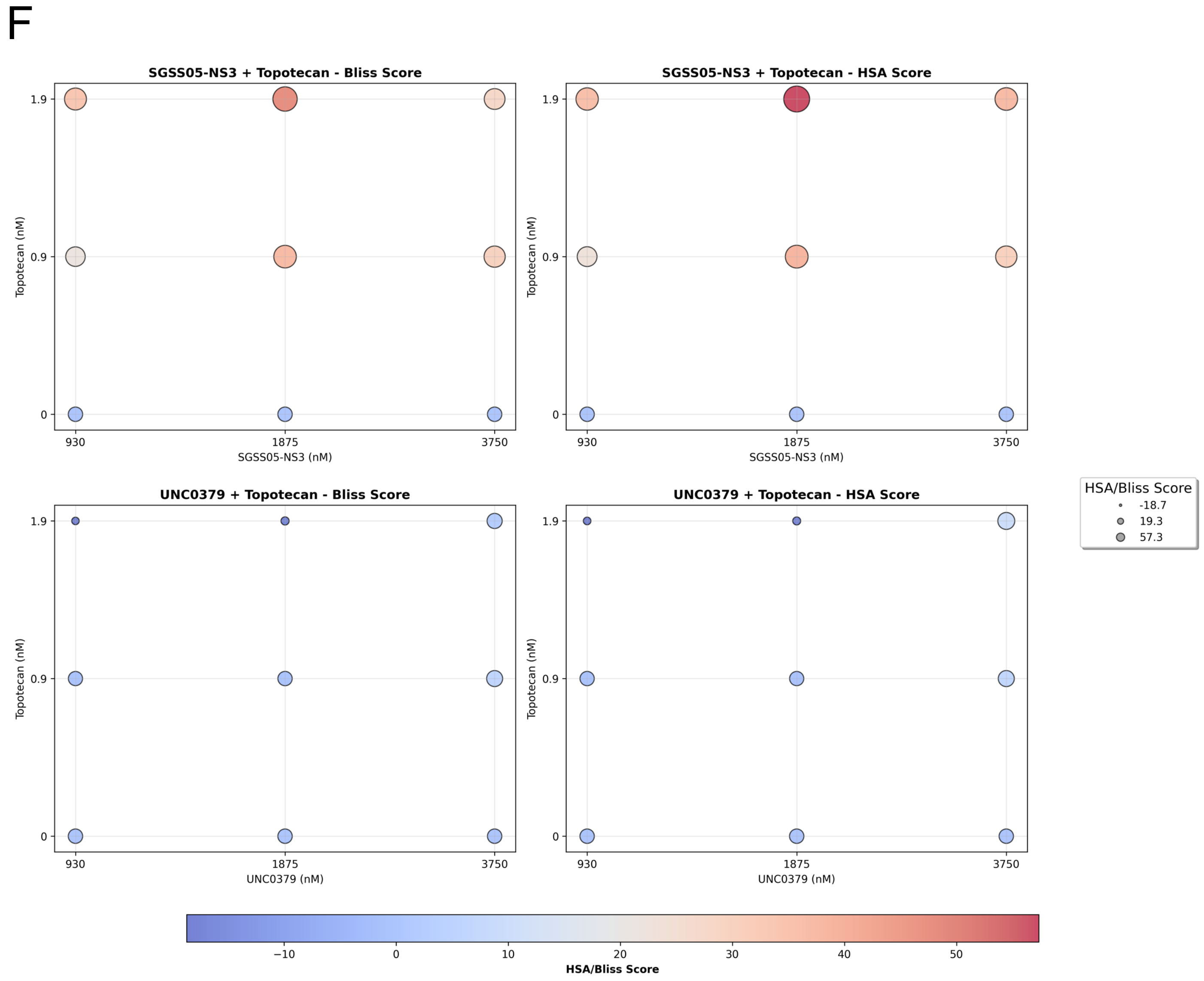
F
SGSS05-NS3 + Topotecan - Bliss Score
SGSS05-NS3 + Topotecan - HSA Score
UNC0379 + Topotecan - Bliss Score
UNC0379 + Topotecan - HSA Score
Topotecan (nM)
1.9
0.9
0
930
1875
3750
SGSS05-NS3 (nM)
UNC0379 (nM)
HSA/Bliss Score
-18.7
19.3
57.3
−10
0
10
20
30
40
50
HSA/Bliss Score

Figure S7

Figure S7, related to Figure 7

Topotecan, a topoisomerase inhibitor, impairs NB cell growth alone and in combination with SETD8 inhibitor, UNC0379 or SGSS05-NS3 compound, enhances the anti-proliferative effects

A) Average IC_{50} values of Topotecan alone calculated based on the cell confluence values of 3 biological replicates in the indicated NB cell lines, at the indicated time points (average of 3 biological replicates). n.d.=not determined.

B, C) An illustrative experiment showing Topotecan *in vitro* effects on cell viability at indicated time and concentrations in MYCN-amp NB cells IMR32 (B) and KCNR (C). Bars show the average of three replicates ± SD.

D, E) Heatmaps showing the percentage of cell confluence upon different concentrations (nM) of Topotecan and SGSS05-NS3 (D) or UNC0379 (E), in MYCN-amp NB cells KCNR at 96 hours. Cell proliferation was measured by Incucyte cell confluence assay.

F) 2D scatter plots showing Bliss and HSA synergy scores for SGSS05-NS3 (*upper panels*) and UNC0379 (*lower panels*) in combination with Topotecan in MYCN-amplified NB cells IMR32, at the indicated concentrations at 96 hr. Each point represents a specific drug combination, with the x-axis indicating SETD8 inhibitor concentration (nM) and the y-axis indicating Topotecan concentration (nM). Dot color reflects the synergy score (Bliss or HSA), according to the color scale (dark blue: antagonism, light blue/white: additive, red: synergy). Dot size is proportional to the synergy score value according to the size legend.

Table S3, related to Figure 7

List of bliss values obtained from the combination of different concentrations of Topotecan (nM) and SETD8 inhibitor, SGSS05-NS3 or UNC0379 (µM) in NB cells (NBLS, SY5Y, SAN, KCNR and IMR32) at the indicated time points.

Provided as an Excel file.
